# Low-Molecular-Weight Metabolites from Diatoms: Structures, Biological Roles and Biosynthesis

**DOI:** 10.3390/md13063672

**Published:** 2015-06-09

**Authors:** Valentin Stonik, Inna Stonik

**Affiliations:** 1 Laboratory of Chemistry of Marine Natural Products, G.B. Elyakov Pacific Institute of Bioorganic Chemistry, FEB RAS, Vladivostok 690022, Russia; E-Mail: stonik@piboc.dvo.ru or stonikiv@mail.ru; 2 Far Eastern Federal University, Sukhanova Str. 8, Vladivostok 690950, Russia; 3 Laboratory of Ecology of the Shelf Communities, A.V. Zhirmunsky Institute of Marine Biology, FEB RAS, Vladivostok 690041, Russia

**Keywords:** diatoms, lipids, oxylipins, sterols, isoprenoid hydrocarbons, pigments, domoic acid, miscellaneous

## Abstract

Diatoms are abundant and important biological components of the marine environment that biosynthesize diverse natural products. These microalgae are rich in various lipids, carotenoids, sterols and isoprenoids, some of them containing toxins and other metabolites. Several groups of diatom natural products have attracted great interest due to their potential practical application as energy sources (biofuel), valuable food constituents, and prospective materials for nanotechnology. In addition, hydrocarbons, which are used in climate reconstruction, polyamines which participate in biomineralization, new apoptotic agents against tumor cells, attractants and deterrents that regulate the biochemical communications between marine species in seawaters have also been isolated from diatoms. However, chemical studies on these microalgae are complicated by difficulties, connected with obtaining their biomass, and the influence of nutrients and contaminators in their environment as well as by seasonal and climatic factors on the biosynthesis of the corresponding natural products. Overall, the number of chemically studied diatoms is lower than that of other algae, but further studies, particularly those connected with improvements in the isolation and structure elucidation technique as well as the genomics of diatoms, promise both to increase the number of studied species with isolated biologically active natural products and to provide a clearer perception of their biosynthesis.

## 1. Introduction

Diatoms (Bacillariophyta) are a division (or phylum), representing a widespread and ecologically important group of microalgae [1,2,3]. Diatoms inhabit both sea and freshwaters and can be found in soils and even in aerosols. In addition to pelagic and benthic forms, some groups of epiphytic diatoms, inhabiting the surface or associated with other micro- or macroorganisms, are also well known. These low plants probably originated in the early Jurrasic period or before, but not less than 185 millions years ago. Today about 100,000 extant species are thought to exist in both marine and freshwaters, although only 12,000 species have so far been described [2]. Usually, diatoms are non-motile and live suspended in the sunlit surface layer of seawater at the action of wind and sea currents. They can regulate their buoyancy via the biosynthesis of lipids, which are more lightweight than other chemical constituents of these organisms. Traditionally, diatoms are divided into two subgroups: centric diatoms, which are radially symmetric (Centrales) and pennate diatoms which are bilaterally symmetric (Pennales) [1,2,3]. In contradistinction to higher plants, diatoms are believed to be derived from a serial secondary endosymbiosis and contain red algal-originated chloroplasts. Diatom plastids are surrounded by four membranes, the outer two representing the plastid endoplasmic reticulum (interpreted as the host vacuole membrane and eukaryotic endosymbiont plasmalemma) and two membranes of the organelle itself [3].

As heterokonts, these microalgae include both autotrophs (majority of species) and obligate heterotrophs or at least the species living as heterotrophs in the absence of light. Due to their short life cycle and rapid turnover, diatoms are the most important producers of primary production of the world’s oceans and contribute significantly to global carbon fixation. Being, as a rule, the main constituents of phytoplankton, diatoms represent an important part of marine animals’ food and are one of the bases of food webs in the marine environment. The decomposition of diatoms over millions of years, with their frustules sinking to the bottom of the sea, has formed tremendous amounts of sediments such as diatomite or diatomaceous earth [3]. These materials have found commercial uses as mineral filters, abrasives, sorbents, anti-caking agents, insulation materials and so on. However, diatoms are a rich source of not only minerals but also of valuable organic natural products (natural bioactive compounds), and herein we present a short review of the structures and properties of these types of compounds. To the the best our knowledge, a review of all the main groups of low-molecular-weight natural compounds, isolated and structurally studied from diatoms, is so far missing in the literature. Herein, we discuss the results obtained by studies on these natural products, according to the order of the state of knowledge and multiplicity of some or other structural groups, starting with lipids.

## 2. Lipids

### 2.1. Fatty Acids and Triacylglycerols

As known, lipids as storage products in diatom algae are transferred via food webs into marine animals, which do not produce polyunsaturated fatty acids themselves. Due to the great significance of diatom lipids in the feeding of many marine organisms such as edible mollusks and fish [4], some species of these microalgae have been investigated for their lipid compositions starting in the 1960s. Ackman, a Canadian chemist was the best known for his pioneering work on marine oils and lipids as well as on omega 3 fatty acids, including those from microalgae [5,6,7]. His group used capillary gas-liquid chromatography and developed methods for fatty acid analysis, which are now used worldwide. The group also elaborated the corresponding methods to produce omega-3 fatty acid-containing oils capsules for medicinal applications [8].

**Figure 1:**
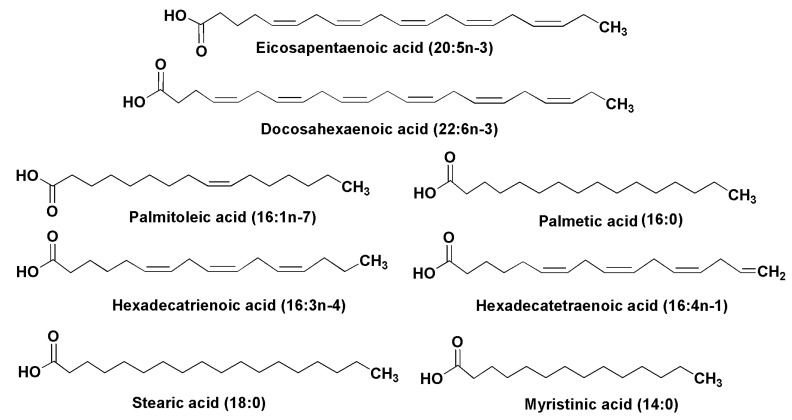
Some fatty acids from diatoms.

In many cases, the lipids of microalgae carry characteristic fatty acids (Figure 1).

Essential polyunsaturated fatty acids (PUFA), including eicosapentaenoic (20:5*n*-3) (EPA) and docosapentaenoic (22:6*n*-3) (DHA) acids, have been found in lipids from 14 species of diatoms [9]. Viso and Marty have found EPA and palmitoleic (16:1*n*-7) acids as prominent fatty acid constituents of diatoms in studies on 28 marine microalgae [10]. Hexadecatrienoic (16:3*n*-4) and hexadecatetraenoic (16:4*n*-1) acids were identified in lipids from the diatom *Skeletonema costatum*, one of the most common species of phytoplankton, along with other above-mentioned acids [4,11]. The last fatty acid seems to be unique and was encountered predominantly in diatoms. A similar composition with an additional prominent fatty acid constituent, palmitic acid (16:0), was established from another widely distributed diatom, *Phaeodactylum tricornutum* [12,13]. Freshwater diatoms such as *Synedra acus* also contain eicosapentaenoic acid (up to 20% of the fatty acid mixture). Myristinic acid (14:0) is widely distributed in diatoms. In a culture of *Synedra acus* from Lake Baikal, in addition to EPA-containing lipids the alga produced lipids with 14:0, 16:0, 18:0, 16:1, 16:2, and 22:6 fatty acids, and palmitic and stearic acids were accumulated in the exponential growth phase [14,15]. In many groups of diatoms myristinic and palmitic acids were accumulated in the lag and logarithmic phases of growth in microalgae cultures, while eicosapentaenoic acid was accumulated in the stationary phase. In comparison with other algal taxa, diatoms are highly enriched with the C14 fatty acids and contain fewer stearic and other C18 fatty acids, while EPA was found to be the most characteristic fatty acid in lipids of diatoms. Rare C24–C28 polyunsaturated fatty acids were also found in diatoms and other microalgae [16,17].

**Figure 2:**
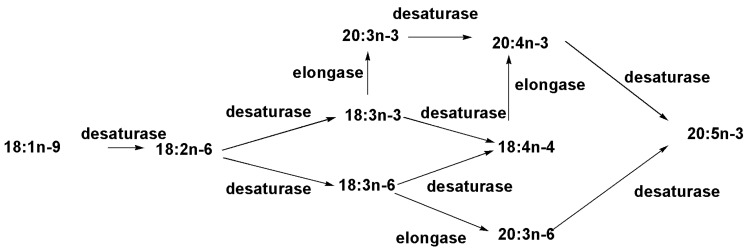
The simplified scheme of the biosynthesis of EPA in PC of *Phaeodactylum tricornutum* [18].

Biosynthesis of omega-3 fatty acids in diatoms has attracted much attention because the lipids containing these acids are used as dietary supplements for humans and there is an increasing scale of industrial production and utilization in the health service. Although the supplements are produced from fish oils, diatoms have proved to be genuine biological sources of the corresponding lipids in the marine environment. In their studies on the biosynthesis of polyunsaturated fatty acids in the pennate diatom *Phaeodactylum tricornutum*, Arao and Yamada [18] carried out experiments with radioactive [1-^14^C]-oleic acid (18:1*n*-9) and observed not only an accumulation of radioactivity from this *de novo* biosynthesized acid in the labeled EPA, but also in the intermediates such as [^14^C]-(18:2*n*-6), [^14^C]-(18:4*n*-3), [^14^C]-(20:4*n*-6), and [^14^C]-(20:4*n*-3). The application of a series of other labeled precursors and inhibition of biosynthesis by different compounds enabled the conclusion to be drawn that arachidonic acid in diatoms is biosynthesized by a network of pathways that contains two routes from 18:2*n*-6, as well as two routes that pass through 18:3*n*-3 and two routes through 18:3 as intermediates followed by elongation to the corresponding 20:4 fatty acids. Further desaturation gives EPA. Therefore, it was shown that EPA in *P. tricornutum* is the end-product of fatty acid biosynthesis, which is realized through classical ω-3 and ω-6 pathways and some alternative pathways with the inclusion of intermediates of both these biosynthetic directions (schema in the Figure 2). The nucleotide sequence of this alga genome confirmed the presence of genes, encoded desaturases and elongases of fatty acid biosynthesis [19].

*Fistulifera* sp., strain JPCC DA 0580 is another diatom, studied in details for the biosynthesis of fatty acids in this group of microalgae. Being well investigated by multi-omic techniques, including genome [20], transcriptome, lipidome and proteome [21] analyses, the alga was screened for candidate genes involved in lipid metabolism, using several bioinformatics programs [21]. It was established that desaturases and elongases providing the EPA biosynthesis, in which ω-3-desaturase expressed in the chloroplast of *Fistularia,* where the final stage of the EPA biosynthesis is performed. The studies on the detailed fatty acid profile by GC-MS method suggested that EPA is biosynthesized only through the ω-6-pathway in this microalga [22].

Generally, in recent years, in studies on the fatty acids of microalgae the main focus has been on the ω-3 series of polyunsaturated fatty acids, such as EPA and DHA, and on the search for new biological sources of these compounds and the conditions required for increasing their content in cultivated diatoms. Some derivatives of fatty acids such as long-chain alcohols were also found in diatoms. For example, 18:1 fatty alcohol was identified in the diatom *Skeletonema costatum* [23].

Diatoms can effectively biosynthesize and accumulate triacylglycerols (TAGs), rich in energy and executing a storage function, which in addition due to extra buoyancy is necessary to counteract the drowning effect of their heavy silica cell walls. Being main storage lipids, TAGs represent one of the major lipid classes of highly productive and fast-growing diatoms. As a consequence, these microalgae have great potential as a biofuel source without competing with arable land products. Moreover, the biosynthesis of these lipids can be induced by environmental stresses [24,25]. The TAGs are formed as a result of photosynthesis in the light, stored in cytosolic bodies and reutilized during biosynthesis of polar lipids in the dark, providing specific acyl groups within them [26]. Although a wide variety of the above-mentioned fatty acids has been found in TAGs of diatoms, many of the lipids from predominant algae were enriched by C14 and C16 fatty acids [27,28]. A range of 4% to 32% of the 14:0 acid of total fatty acid content was reported in diatoms [24]. Palmitoleic acid (16:1) was found to be the predominant monoenoic acid in some diatoms, and the hypothesis was proposed that TAGs of these microalgae, containing mainly C16 fatty acids in the *sn*-2 position (as is characteristic of “prokaryotic-type lipids”), are mainly derived from lipids in chloroplasts [29] in contrast with so-called “eukaryotic-type lipids”, which possess C18 (C20) fatty acids in these positions [30]. However, the relationship between biosynthetic pathways in diatoms is more complicated than in prokaryotes and higher plants, because the chain elongation reaction from C18 to C20 fatty acids takes place only in the biosynthesis of EPA [28].

Stress conditions such as limitation of nutrients, increased temperature *etc.* allow significant increase in both the content of total lipids and TAGs in diatom cultures. For example, the average lipid content in diatoms was about 23% of dry cell weight, but this might be increased almost twofold as a result of the stress caused by nutrient-deficient conditions [31]. The content of TAGs in *Cyclotella crypta* and other diatoms was reported to be about 60%–70% of neutral lipids on dry and ash-free weight in Si-deficient cultures compared with about 30% in cultures repleted with Si [24]. During the transformation of diatom cells, connected with overexpression of glycerol-3-phosphate dehydrogenase, the increasing dihydroxyacetone content, obtained as a result of glycolysis, and promotion of the conversion of the latter into glycerol-3-phospate were observed. As a result, the level of 16- and 18-monounsaturated acids in *P. tricornutum* was significantly increased [32].

### 2.2. Polar Lipids

**Figure 3:**
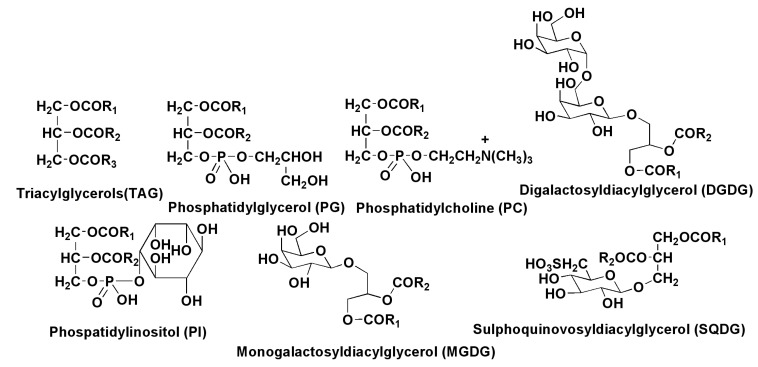
Main lipid constituents of diatoms.

The polar lipid fraction from diatoms mainly consists of monogalactosyldiacylglycerol (MGDG), digalactosyldiacylglycerol (DGDG), sulfoquinovosyldiacylglycerol (SQDG), phosphatidylcholine (PC), phosphatidylinositol (PI), phosphatidylglycerol (PG), and others, usually minor constituents (Figure 3).

*P. tricornutum* contains large amounts of EPA in MGDG, PC, PG, DGDG, and myristinic acid located mainly in the *sn*-1, but not the *sn*-2 position in all the polar lipids [28]. Understanding of glycerolipid biosynthesis in the alga *Fistularia solaris* was recently achieved using a lipidomic approach based on ESI-Q-TRAP and MS/MS analyses. Palmitic and stearic acids as well as their monounsaturated derivatives are *de novo* biosynthesized in chloroplasts, but the biosynthesis of glycerolipids proceeds in the endoplasmatic reticulum, located between the outermost and second outermost membranes of the chloroplast. In this compartment, all glycerolipids, with the exception of PC, are acylated by C16 fatty acids by the “prokaryotic pathway” at the *sn*-2 position, while phosphatidylcholines are partly acylated by the “eukaryotic pathway” with the incorporation of C18 acids into the *sn*-2 position. Further, C18 fatty acid residues are transformed into polyunsaturated C18 and C20 derivatives, including EPA, at the action of desaturases and elongases. This PC-based acyl-edition and head group exchange is essential for the incorporation of EPA into other glycerol lipids, including TAGs, glyco- and phospholipids [33].

**Figure 4:**
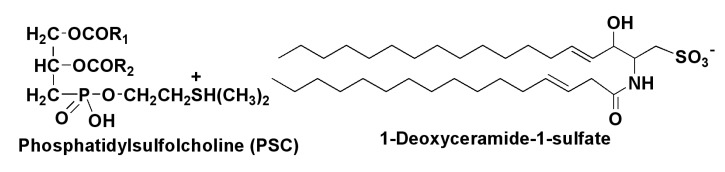
Some sulfur-containing lipids from diatoms.

Some lipids in diatoms are presented as rare sulfoforms (Figure 4). Besides sulfoquinovosyldiacyl-glycerol, there are 1-deoxyceramide-1-sulfate and phosphatidylsulfocholine, discovered along with 24-methylene cholesterol sulfate from the marine non-photosynthetic diatom *Nitzschia alba* in 1978 [34]. Phosphatidylsulfocholine was detected alone in *N. alba*, but it was found in *N. angularis*, *Cylindrothecha fusiformis*, *Navicula pelliculosa* and *Phaeodacylum tricornutum* in a mixture with the usual phosphatidylcholine [35]. Biosynthetic experiments showed that sulfur is incorporated into the sulfolipids from the sulfur-containing amino acids methionine, cysteine, and cystine [36].

Some lipids show interesting biological activities, particularly with regard to unsaturated fatty acids found from diatom lipids. For example, 18:1 and 18:2, which are usually minor components of fatty acid fractions, show antimicrobial properties [37]. Polyunsaturated ω-3 acids, such as eicosapentaenoic acid, are not only important constituents of numerous food additives, used for medicinal and prophylactic purposes, but also influence the marine environment. Being incorporated into epilithic diatom biofilms, arachidonic acid is responsible for the toxicity in the *Tamnocephalus platyurus* assay and provides a grazer defense reaction [38].

It is worth noting that compared to the preceding decade there has been a threefold annual growth over the last two to three years in the number of scientific articles about all the groups of diatom lipids. Obviously, this is mainly due to the apparent interest in these microalgae as potential biofuel sources.

It must also be acknowledged that although lipids are one the most studied groups of natural compounds isolated from diatoms, this type of compounds, particularly, concerning some distinct important groups of lipids, for example, sphingolipids, has not as yet been investigated from the absolute majority of species.

## 3. Oxylipins

Diatoms are a rich source of oxylipins [39], oxygenated derivatives of fatty acids and products of the subsequent transformations of these derivatives. They are formed upon the oxidation of fatty acids to hydroxyperoxides by the action of iron nonheme enzymes lipoxygenases (LOXs), which add molecular oxygen to the carbon chain of fatty acid and are classified according to the position of oxygen addition. The succeeding action of hydroperoxide lyases (HPLs) may lead to compounds having a shorter carbohydrate chain, so-called volatile oxylipins. In contrast, oxylipins containing C16–C22 carbon atoms oxylipins are called non-volatile compounds. Oxylipins were proved to be both ecological and physiological mediators. Usually, all special lipoxygenases trigger a cascade process, leading to a corresponding set of products that play an important role in allelopathic competition, predator–prey interactions and prey capture [39,40,41].

It is known that productive regions of the ocean are characterized by seasonal blooms of diatom phytoplankton [42]. They not only provide plenty of food for different consumers, including copepods and fish, but also cause some inhibitory effects against predators. On the basis of laboratory experiments it has been shown that, although dominant zooplankton grazers such as copepods feed extensively on diatoms, the hatching success of their eggs is seriously impaired and only 12% of copepod eggs hatch compared with 90% in post-bloom conditions [42]. Polyunsaturated aldehydes (PUAs), isolated from the diatom *Thallasiosira rotula* after its blooming, were proposed as the compounds responsible for this biological activity. Moreover, it was shown that these compounds arrest embryonic development not only in copepods, but also in sea urchins and ascidians, and demonstrate antiproliferative and apoptotic effects on human carcinoma tumor cells [42]. However, this work opened up international debates and soon it was noted that “paradoxically, high diatom abundance could limit secondary production”. In their studies on 12 globally distributed areas, where diatoms dominate the phytoplankton assemblage, Irigoien, X. *et al.* [43] did not observe a negative relationship between PUAs and copepod egg hatching success. They predicted the effect of PUAs rather through the benefit diatom competitors, but not due to anti-grazers’ defense against the copepods. In addition, PUAs induce a structural defense (thicker cell walls) to resist zooplankton grazing when phosphorus and nitrogen are limited during the blooms.

**Figure 5:**
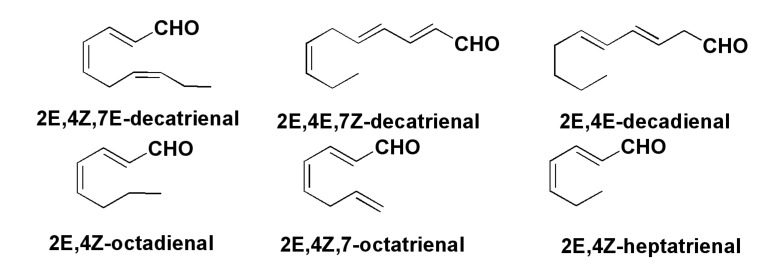
Polyunsaturated aldehydes (PUAs)-products of oxylipinic biogenesis.

Numerous studies on bioactive aldehydes (Figure 5), products of oxylipin biogenesis, have confirmed that these compounds inhibit, despite their very simple structures, the development of embryos of different marine invertebrates such as sea urchins, starfish, polychaetes, and ascidians, reducing hatching success and larval recruitment. Therefore, these compounds are teratogenic against grazers and deleterious for planktonic species [39]. Recently, 51 species of diatoms were examined for PUA-release. About 38% of the cultivated strains released α,β,γ,δ-unsaturated aldehydes upon cell disruption in concentrations from 0.01 to 39.8 fmol per cell [44]. Thus, a wound-activated mechanism for the production of deterrent oxylipin toxins probably induces reproductive failure in grazing copepods and other invertebrates, including their juvenile forms. This effect strongly depends on abiotic factors such as nutrients and silica availability, diatom growth stage and season. Vardi *et al*. [45] reported that seawater diatoms themselves can detect the presence of decadienal-like PUAs, which induce the inhibition of cell growth, the damage and cell death via calcium signaling and a nitric oxide pathway. This may represent chemical control of diatom bloom dynamics. Thus, PUAs are “infochemicals” that trigger different responses in marine biosystems.

**Figure 6:**
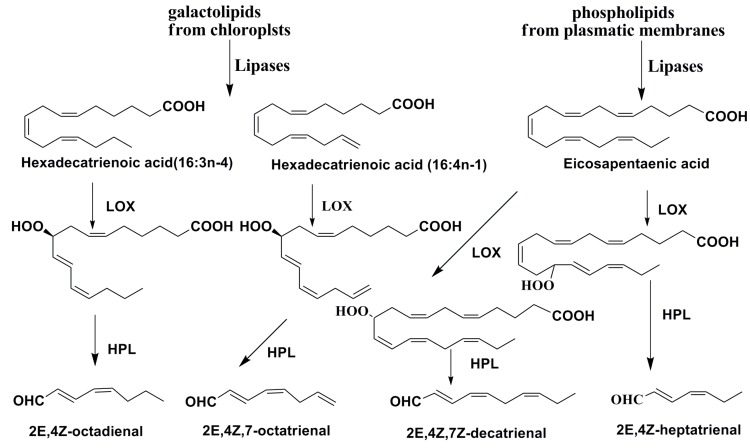
Biosynthesis of PUAs in diatoms *T. rotula* and *S. costatum* [48,49,50,51,52].

Several additional PUAs, given in Figure 5 were identified by Italian scientists in chemical studies on the biosynthesis of PUAs in the diatom *Skeletonema costatum* [46]. Using labeled precursors, they showed that both C16 and C18 fatty acids of galactolipids from cultures of *Skeletonema* *costatum* and *Thalassiosira rotula* [47] as well as eicosapentaenoic acid from phospholipids of these algae are transformed into PUAs. Galactolipids at the action of lipases give both C16 and C18 fatty acids, while phospholipids are mainly a source of eicosapentaenoic acid. Phospholipase PLA_2_ probably plays a key role in this process. The action of LOXs leads to the introduction of hydroperoxy groups in these substrates. It is of special interest that these enzymes keep their activities in seawater for some time after the disruption of diatom cells [48]. Consequent reactions with polar lipids give different deterrent PUAs. Thus, lysis of diatom cells induces the formation of a variety of lipid compounds, in which the main role is played by polyunsaturated fatty acids and enzymes responsible for their liberation from lipids and further transformation into precursor oxylipins and then into PUAs (Figure 6).

Lipoxygenases (LOX), catalyzing the oxygenation of all the main unsaturated fatty acids in diatoms usually give a variety of important oxylipins, participating in signal transduction. According to the initial fatty acid, these compounds may be divided into groups of C-16, C-20, and C-22 derivatives. Moreover, the specificity of a LOX provides the precise incorporation of a hydroperoxide group into the preset positions in the substrate, and, therefore, the directions of further transformations.

The LOX-initiated conversion of 16:3 and 16:4 polyunsaturated acids at the absence of C-18 PUFA-derived oxylipins differentiates the oxylipin biosynthesis in diatoms from that in other algae, higher plants and animals.

**Figure 7:**
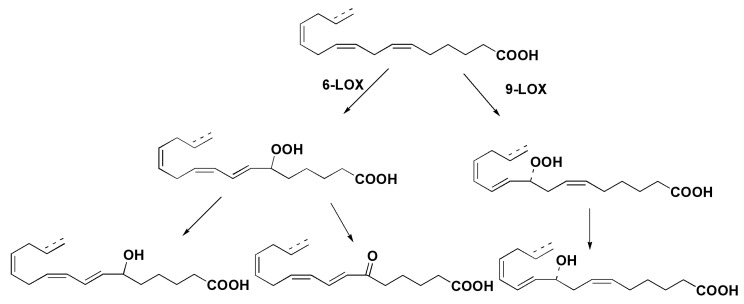
Hypothethic scheme of the origin of oxylipins in *T. rotula* [52].

Italian scientists described unprecedented series of C16 oxylipins along with C20 PUFA derivatives from the marine diatom *Thalassiosira rotula* [52]. Structure elucidation, including absolute configuration of the major alcohol derived from the corresponding hydroperoxide, showed the occurrence of 9*S*-lipoxygenase in living cells of this microalga. Another direction includes the participation of 6-lipoxygenase. Substrates of these enzymatic transformations were proposed to be 16:3*n*-6 fatty acid and some other fatty acids from galactolipids of chloroplasts. Structures of oxylipins obtained are given in Figure 7.

Some diatom species do not produce PUAs, for example, *Skeletonema pseudocostatum*. However, it has a strong effect on the reproductive parameters of the copepod *Temora stylifera* and produces a series of oxylipins, such as (5*Z*,8*Z*,11*Z*,13*E*,15*S*,17*Z*)-15-hydroxy-5,8,11,13,15,17-eicosapentaenoic acid (15*S*-HEPE), 13,14-13*R*-hydroxy-14*S*,15*S*-*trans*-epoxyeicosa-5*Z*,8*Z*,11*Z*,17*Z*-tetraenoic acids, and 15-oxo-5*Z*,9*E*,11*E*,13*E*-pentadecatetraenoic acid, suggesting that their effect may really be dependent on the production of non-volatile oxylipins [53].

Using tandem mass spectrometry, nuclear magnetic resonance (NMR) and radioactive probes, structures of oxylipins and growth-dependent modulation of their biosynthesis were studied in the pennate diatom *Pseudo-nitzschia delicatissima*. Three oxylipins 15*S*-HEPE, 15-oxo-5*E*,9*E*,11*E*,13*E*-pentadecatetraenoic acid, and 13,14-treo-13-hydroxy-14*S*,15*S*-*trans*-epoxy-5*Z*,8*Z*,11*Z*,17*Z*-tetraenoic acid, were found to be products of three putative pathways, triggered by eicosapentaenoic acid-dependent 15*S*-lipoxygenase [54].

**Figure 8:**
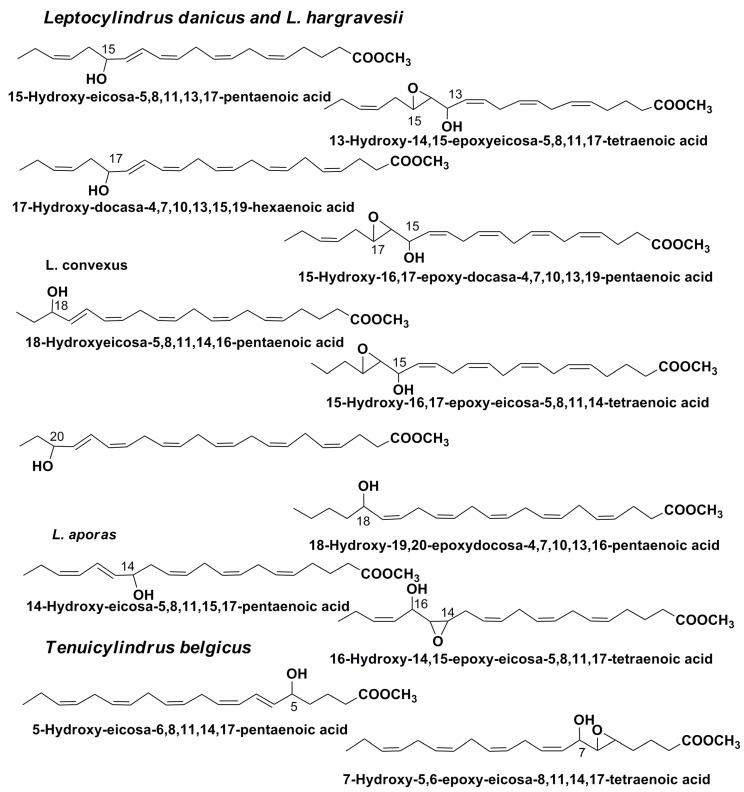
Some methyl esters of oxylipins from diatoms, belonging to the family Leptocylindraceae.

Additionally, a series of other oxylipins, including derivatives of docosahexaenoic acid, were identified by GC/MS as methyl esters in different Leptocylindraceae diatom species [55] (Figure 8). The study gave additional information concerning the distribution of lipoxygenases in diatoms. Some identified oxylipins may be considered as products of the transformation of EPA acid in the interaction with 18-LOX, 15-LOX, 14-LOX, and 5-LOX. In addition, oxylipins derived from DHA in the action on this acid of 17-LOX and 20-LOX were found. It was suggested that single enzymes may be responsible for the parallel metabolism of both EPA and DHA fatty acids [55]. It was also shown that distinct oxylipin patterns in related species can be indicative of even small genetic differences. The finding of DHA derivatives among oxylipins increases their known diversity. Moreover, unique combinations of different compounds belonging to this chemical class can reflect their species identity. This is particularly important because the problem of distinguishing diatom species is now known to be a key problem in the taxonomy of diatoms, after evidence was found showing that many species, defined as cryptic, are not distinguishable morphologically.

Thus, the presence of diverse LOXs was documented in different species of diatom microalgae. These enzymes act on substrates belonging to C-16, C-20 and C-22 polyunsaturated fatty acids together with other enzymes, not only hydroperoxide lyases, but also epoxy alcohol synthases, peroxidases, and different cyclases [39,40,55]. In addition, the action of LOXs sometimes accompanies ulterior non-enzymatic transformations.

**Figure 9:**
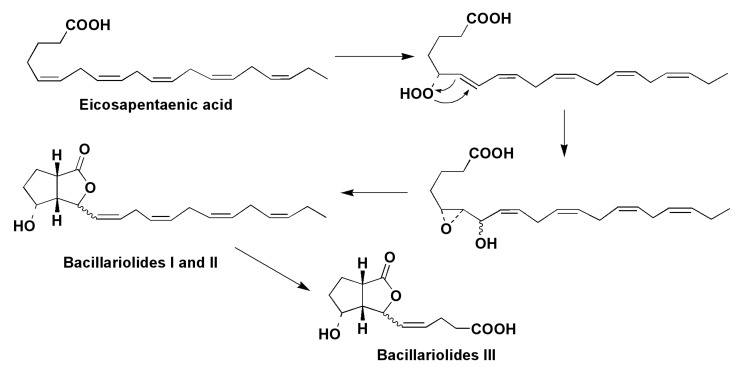
Proposed biogenesis of bacillariolides I and II in the diatom *Pseudo*-*nitzschia pungens* [58].

The marine diatom *Nitzschia pungens* (*=Pseudo-nitzschia pungens*), known as the causative agent of amnesic shellfish poisoning, contains a series of new metabolites belonging to the oxylipin structural class, which are biosynthesized by several enzymes, beginning with the action of 5-LOX on eicosapentaenoic acid. Two epimeric oxylipins, so-called bacillariolides I and II, and bacillariolide III, derived from the first of them, were obtained from both field-collected and cultured cells [56,57]. The structure of bacillariolide I was confirmed by X-ray analysis of its crystalline camphenic acid derivative [58]. It was suggested that bacillariolides are formed at the cyclization of epoxyalcohols, well known as rearrangement products of diene-hydroperoxides from lipoxygenase metabolism (Figure 9).

**Figure 10:**
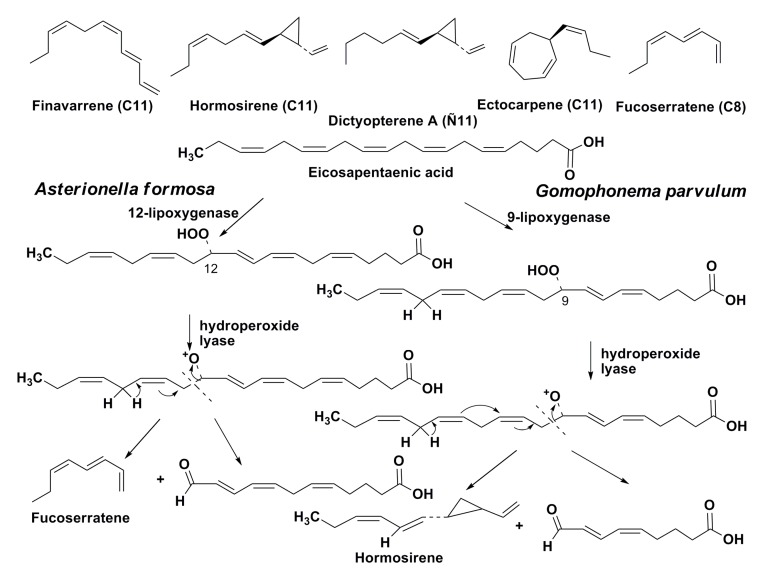
Volatile hydrocarbons found in diatoms, and biosyntheses of homosirene and fucoserratene in the freshwater diatoms.

Some marine and freshwater diatoms produce volatile alicyclic olefins [59], which are possibly phytoplanktonic pheromones. It is of particular interest that the same compounds were earlier found in brown algae. The production of ectocarpene, previously known as a pheromone of brown algae, by two species of diatoms, namely the marine diatom *Skeletonema costatum* and the freshwater diatom *Lethodesmium undulatum*, was confirmed in 1986 by Derenbach and Pesando [60]. Later Wendel and Juttner [61] established that some freshwater diatoms synthesized in a lipoxygenase-mediated manner unsaturated and alicyclic hydrocarbons, including those known from brown algae C_11_ pheromones such as finavarrene, hormosirene, dictyopterene A, and ectocarpene (Figure 10). These compounds in brown algae induce spermatozoid release and provide a gradient of attractant leading to the signaling of fertile female gametes. The release occurs within 8–12 s and it is one of the fastest signal responses in plants. The biological functions of these pheromones in diatoms have been studied less than those of brown algae.

Biosynthesis of hormosirene in the freshwater diatom *Gomphonema parvulum* proceeds from eicosapentaeinic acid by oxidative degradation at the action of 9-lipoxygenase and hydroperoxide-lyase [62], while another pheromone, fucoserratene, is similarly biosynthesized in the freshwater diatom *Asterionella* *formosa* at the action of 12-lipoxygenase and the corresponding hydroperoxide lyase [63] (Figure 10). Therefore, these compounds in diatoms are also products of oxylipin biogenesis. The oxo acids, which are formed together with fucoserratene at its biosynthesis, seems to demonstrate defensive properties against amphipods, which usually feed on diatoms [64]. The biological functions of these pheromones in diatoms have not been yet studied and is one of the unresolved problems of diatom physiology.

The number of known representatives of products of the lipoxygenase biosynthetic pathway is rapidly growing. It is now evident that these compounds are among the most characteristic metabolites in diatoms. Their biological functions are significantly involved in inter- and intraspecies communications, and their influence on biochemical processes within their producers remains insufficiently studied.

## 4. Sterols

Sterols are important membrane constituents of eukaryotic organisms, which partly determine the stability, permeability and barrier properties of biomembranes. Diatoms, as microorganisms that contribute greatly to the primary production in oceans and other aqueous basins (approximately one fifth of the world’s primary production is related to diatoms) and one of the major sources of organic matter, contain large amounts of some characteristic sterols, which may also be considered as biomarkers of the food web. On the other hand, these microalgae became significant in number about 35–40 millions years ago and from that time formed fossils, which enabled the use of sterols from fossils in paleocenographic studies to reconstruct the compositions of microalgal communities for different geologic time ranges depending on existing climatic peculiarities [64,65].

**Figure 11:**
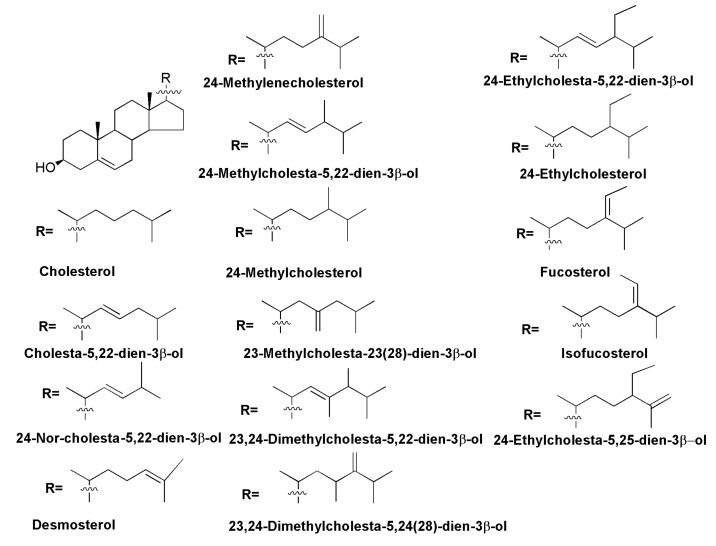
Sterols of Δ^5^-series from diatoms.

The presence of sterols was found in all the main taxonomic groups of diatoms, including the oldest radial centrics, other centrics, araphid, and raphid pennates. The most widely distributed sterols belong to the Δ^5^-series with 24-methylencholestrol being a predominant sterol in many marine [66,67] and freshwater diatoms [68], while 24-methylcholesta-5,22-dien-3β-ol (diatomsterol) is also very common and was found as a predominant sterol component in sterol compositions of almost 50% of the studied pennate diatoms [69]. Generally, C_28_ sterols proved to be the main sterols in the majority of diatoms, although some phylogenetic groups contain high a percentage of C_27_ or C_29_ sterols. However, neither 24-methylencholesterol, or 24-methylcholesta-5,22-dienol are accepted as taxonomic markers of diatoms only, since these sterols have also been found in other taxa of microalgae [67]. No sterols were found to be restricted strongly to specific phylogenetic groups of diatoms. However, cluster analysis of sterol mixtures in diatoms is useful for their taxonomy, and it allows us to determine distinct sterol distributions within Bacillariophyta and corresponds to phylogenetic conclusions made on the basis of molecular biology methods. For example, a high abundance of 24-methylenecholesterol is a typical feature of Thalassiosirales, the same distribution of 24-methylcholesta-5,22-dienol may be characteristic of Cymatosirales, diatoms belonging to the genus *Amphora* contain a combination of 24-methylcholesterol and 24-methylenecholesterol as main sterols, while *Attheya* spp. have a significant content of 24-ethylcholesterol [66]. In some studied species one strongly predominant sterol (>90% of the total fraction) was found: cholesterol in *Cylindrotheca fusiformis* and *Nitzschia closterium*, 24-methylenecholesterol in the freshwater diatom *Synedra acus* [69], 24-methylcholesta-5,22-dien-3β-ol in *Stauroneis* *constricta*, *Dickieia ulvaceae,* and *Phaeodactylum tricornutum* [66]. Some diatom species contain rare 23-methyl- and 23,24-dimethylcholesta-5,22-dien-3β-ols [66] (Figure 11).

**Figure 12:**
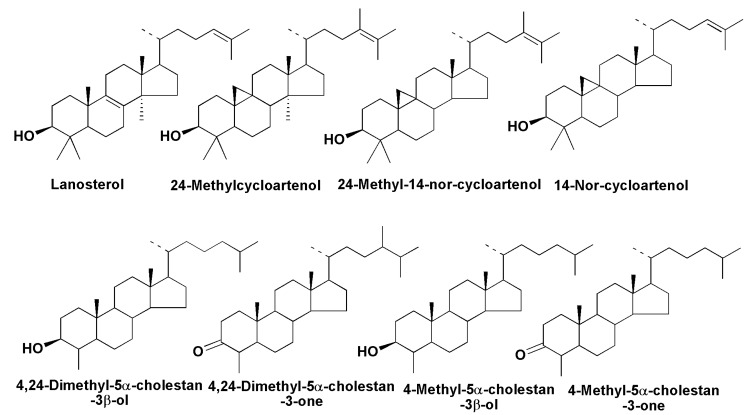
Some compounds related to biosynthesis of sterols *de novo* in diatoms.

Sterols belonging to other series, such as stanols or Δ^7^-sterols, were identified only in a small number of species, mainly as minor constituents. There are several cases of the identification of tetracyclic triterpenoids, which are biosynthetic precursors of sterols. Lanosterol was found in the raphid pennate *Haslea* sp., while together with 24-methyl-14-nor-cycloartenol it was indentified in *Stauroneis simulans*, suggesting that biosynthesis from squalene in Bacillariophyta can proceed via both lanosterol and cycloartenol [66]. (Figure 12). However, it is not clear which taxonomic groups of diatoms biosynthesize sterols via cycloartenol as in higher plants, or whether there are indeed diatoms using lanosterol as an intermediate of this biosynthesis, as in the case of animals and fungi.

Several papers, confirming the *de novo* intensive biosynthesis of sterols in diatoms have been published [70,71,72]. For example, all carbon atoms in the molecule of 24-methylenecholesterol were ^13^C after cultivation of the Baikal diatom *Synedra acus* in NaH^13^CO_3_-containing medium [70]. As was shown by detailed studies, using labeled precursors such as ^13^C-enriched acetate and deuterium-enriched deoxyxylulose as well as specific inhibitions of mevalonate (MVA) and methylerythritol pathways (MEP), the sterols in *Rhizosolenia setigera* are biosynthesized by the MVA pathway, although biosynthesis of some terpenoids in the diatoms *R. setigera* and *Haslea ostrearia* may be realized via both these routes. As a result, it was established that the MVA route is realized mainly in the cytoplasm and membranes of diatoms, in which sterols and some other isoprenoids are localized, while phytol is biosynthesized in chloroplasts where the MEP pathway takes place [72]. Several other compounds, identified in different diatoms, including 3-keto steroid derivatives [66], might be considered as intermediates related to the biosynthesis of sterols *de novo* (Figure 12) or end-products of steroidogenesis, having unknown biological functions.

## 5. Isoprenoid and Other Hydrocarbons

### 5.1. Hydrocarbons, Derived from Fatty Acids

**Figure 13:**
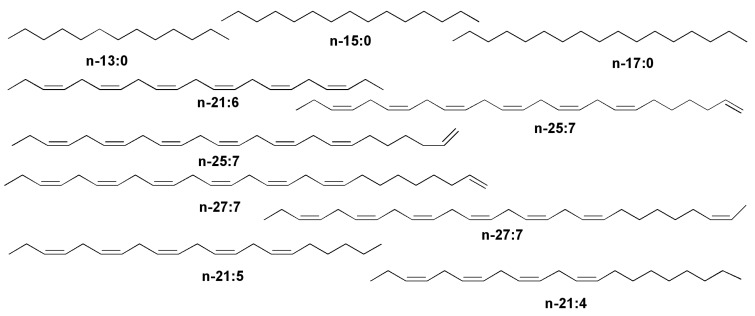
Some hydrocarbons indicated in diatoms.

Several classes of hydrocarbons and related compounds from diatoms are known. The first class includes alkanes such as C17:0 and particularly alkenes found by different groups of scientists (Figure 13). As a rule, these compounds are products of biodecarboxylation of microalgal fatty acids. The *n*-21:6 hydrocarbon, all-(*Z*)-heneicosa-3,6,9,12,15,18-hexaene, found as a main hydrocarbon from photosynthetic diatoms as well as from many other algae is obviously derived from docosahexaenoic acid and it is a product of the corresponding decarboxylation [73,74]. Normal heneicosa-3,6,9,12,15,18-pentaene (*n*-21:5) and the corresponding tetraene were also found in several studied diatoms by Volkman *et al*. [75]. Squalene together with *n*-21:3, *n*-21:4 and *n*-21:5 alkenes, obviously formed by decarboxylation of the corresponding polyunsaturated C22 fatty acids, were indentified in the marine benthic diatom *Pleurosigma strigosum* isolated from coastal Mediterranean sediments [76].

Another group of hydrocarbons are also biosynthesized from fatty acids, but as a result of not only decarboxylation but also the chain elongation that is sometimes followed by the introduction of additional double bonds. For example, along with isoprenoid hydrocarbons, two isomeric *n*-25:7 and two isomeric *n*-27:7 alkenes were identified in the marine diatom *R. setigera*, in which they are obviously formed through the chain elongation [77].

Finally, some hydrocarbons derived from fatty acids found in diatoms could be adsorbed from the environment. For example, it was established that the marine diatom *Cyclotella cryptica* grown in culture was capable of accumulating exogenic hydrocarbons, especially with 16 carbon atoms, from the medium [78].

### 5.2. Isoprenoid Hydrocarbons and Related Compounds

Highly branched C_25_ and C_30_ isoprenoids, widely occurring in bottom sediments and oils [79], with parent structures confirmed by organic synthesis [80], originate from diatoms. In fact, in 1994 Volkman *et al.* [75] reported their occurrence in laboratory cultures of the diatoms *H.* *ostrearia* (C_25_ isoprenoid hydrocarbons) and *R. setigera* (C_30_ isoprenoid hydrocarbons). They found at least seven C_25_ isoprenoids in the culture of *H. ostrearia*. Thus, it was clearly established that diatoms are a major source of this class of unusual hydrocarbons, previously found in marine sediments [69].

Unsaturated isoprene hydrocarbons containing 25 carbon atoms, so-called haslenes, from *H. ostrearia* and other diatoms represent a group of sesterterpenoids with the general characteristic haslane skeleton system. The diatom *H. ostrearia,* identified as a biological source of sedimentary haslenes [81], was cultured in 500 L tanks and a 198 mg non-polar fraction was obtained by extraction with hexane from this non-axenic culture. Separation by haslene mixture using SiO_2_ column chromatography followed by an NMR study led to structures of three highly branched isoprenoids, namely triene, tetraene and pentaene, respectively [81]. Biotesting on some of these alkenes has shown that they are active in decreasing the growth of lung cancer cell lines. As a result, these compounds were patented (Patent GB-9708934.6). In total, more than a dozen dienes to hexaenes were identified from cultures of this alga as well as from some other species, and many of them were also found in bottom sediments [81,82]. An additional highly branched C_25_ isoprenoid triene was later isolated and purified from axenic cultures of the same algal species [83].

The diatom *Rhizosolenia setigera* also contains haslenes, although C_30_-isoprenoids often predominates. For example, haslene pentaene with a *Z*-configuration of 9(10)-double bonds was reported from this species [82] as well as pentaene with all *E*-configurations [84]. A North Atlantic strain of *R. setigera* produces a C_25_ highly branched isoprenoid pentaene in contrast to Australian strains of this species, which produce mainly C_30_ alkenes. It was shown that distribution of haslenes as well as C_30_-isoprenoids in this species depends on the strain and the cultivation conditions [85]. In studies on five strains of this planktonic diatom, Rowland *et al*. [86] detected both haslenes, including hasla-7(20),9*E*,*Z*,23-trienes and hasla-7(20),9*E*,*Z*,13,23-tetraenes, as well as rhizenes (C_30_ compounds) in some strains.

An increase in growth temperature from 18 to 25 °C increased the degree of unsaturation of haslenes and *Z* to *E* isomerization. Unsaturation of haslenes was not changed by the increased salinity, but unsaturation on C_30_ compounds (rhizenes) was decreased [86].

Several new compounds of the haslane series were found in at least four species belonging to the genus *Pleurosigma* [82,87,88].

**Figure 14:**
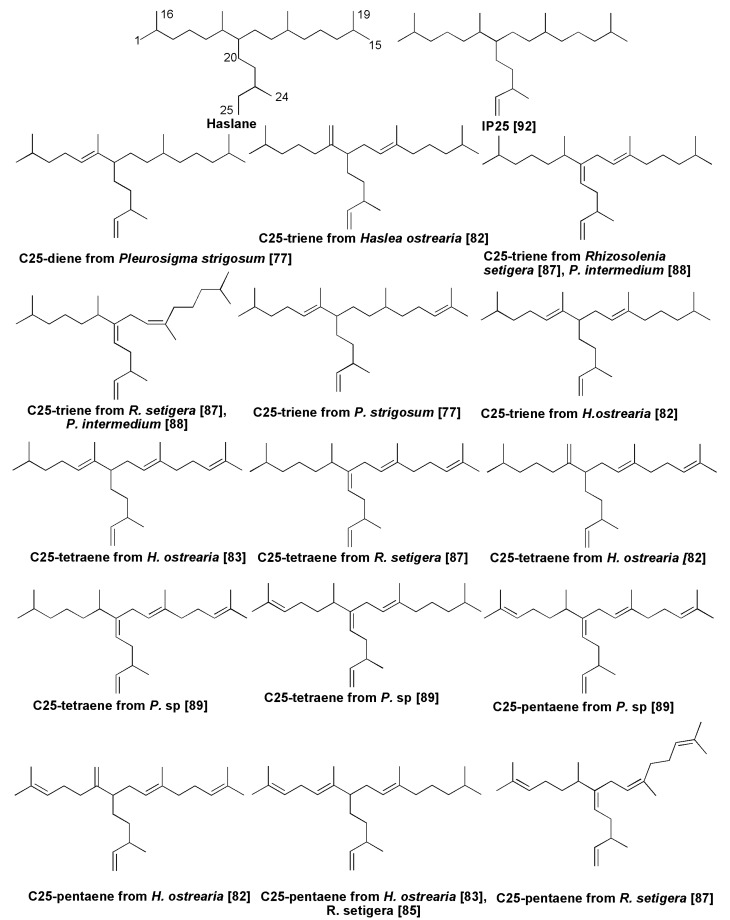
Some C_25_-isoprenoids (haslenes) from diatoms.

**Figure 15:**
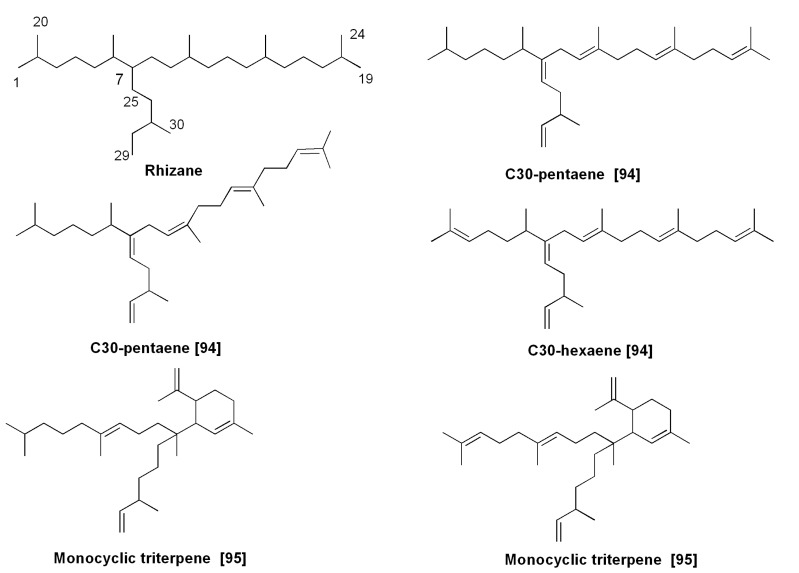
Some C_30_-isoprenoids from diatom *Rhizosolenia setigera*.

Sea ice communities dominated by diatoms such as *Nitzschia stellata*, *Amphiprora* sp. and others in the surrounding Antarctica ice barrier contain a variety of fatty acids and different hydrocarbons, including C_25:2_ isoprenoids [89,90,91,92]. The monounsaturated highly branched isoprenoid (IP25), so-called “ice proxy 25”, (Figure 14) is strictly associated with sea ice and widely distributed in Arctic and subarctic sediments. It was found in sea ice diatoms, such as *Haslea* *crucigera* and *Pleurosigma stuxbergii* var. *rhomboides* from mixed diatom assemblages collected from the Canadian Arctic [93]. IP25 can be used and was partly used as a geochemical marker in the studies on palaeo-climate to reconstruct the sea ice covering in the world’s oceans from the early Pleistocene to decades back [93]. As was mentioned above, C_30_ isoprenoids (triterpenoids) were also found in diatoms, and primarily in *Rhizosolenia setigera* [84,85,86]. They contain the so-called rhizane skeleton system. Among rhizenes, the isoprenoid hexaenes and pentaenes proved to be predominant compounds, and *Z* to *E* isomerization increased, when compared with those of haslenes. Rhizenes were also found in marine sediments. Some examples of diatom rhizenes [94,95] and their natural monocyclic derivatives [95] are given in Figure 15. Monocyclic triterpenes, isolated from the diatom by Ag^+^-HPLC belong to an extremely rare group of terpenoids.

Unexpected data were obtained as a result of the studies on isoprenoid biosynthesis in the diatoms *Rhizosolenia setigera* and *Haslea ostrearia*. By using the ^13^C and ^2^H isotopic labeling technique followed by NMR and MS studies, specific inhibitors of MVA and MEP biosynthetic pathways as well as measurements of ^13^C/^12^C isotope ratios in the studied compounds, a significant difference in biosyntheses of sester- and triterpenoid hydrocarbons and some other diatom metabolites in these algae was determined. Surprisingly, Δ^7(20)^ haslenes and different rhizenes in *R. setigera* were formed by MVA biosynthesis, while the structurally similar haslenes in *H. ostrearia* were biosynthesized by the MEP route. Therefore, triterpenoids are biosynthesized similarly to terpenoids in higher plants, but sesterterpenoids can be made by either the MVA or the MEP routes in a species-dependent manner [72].

Although haslenes and rhizenes are biosynthesized by a limited number of diatom genera, these compounds are strongly characteristic metabolites of these taxa of microalgae. This is more important, due to the above-mentioned dependence of the extent of unsaturation in this series of isoprenoids on the growth of environment temperature. In fact, hasladienes predominate in cultures of diatoms at 5 °C, while haslatrienes and more unsaturated compounds are principally formed at 25 °C [86]. This confirms the validity of the use of diatom isoprenoids from bottom sediments to reconstruct climate changes.

## 6. Pigments

**Figure 16:**
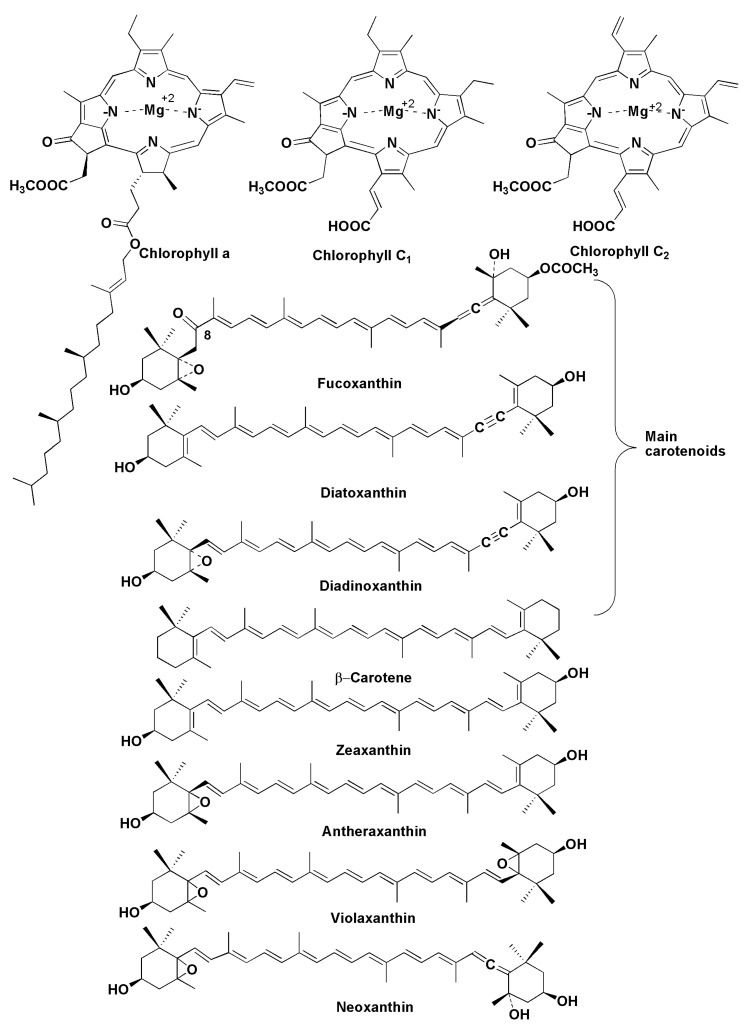
Pigments of diatoms.

Chloroplasts of diatoms contain the following main pigments: fucoxanthin, β-carotene, diatoxanthin, diadinoxanthin [96], and several variants of chlorophylls, including chlorophylls *a*, *c*_2_ and *c*_1_ (Figure 16). For example, studies on 51 strains from 18 species of the genus *Pseudo-nitszchia* showed the presence of chlorophylls *a* and a significant diversity in the content and ratio of chlorophylls *c*_1_ and *c*_2_ between these species [97]. Phytol, a component of chlorophyll *a*, is often present in extracts from diatoms as a free constituent. These pigments play one of the main roles in phytosynthesis, providing light harvesting and photoprotection in diatoms. There is a similarity between pigments of diatoms (Figure 16) and those of brown algae, because chlorophylls *a* and *c* along with the xanthin pigment fucoxanthin are often the main pigments in both taxa, although some participants of the xanthophyll cycle were considered to be quite different in comparison with brown and other algae. In fact, there are predominant pigments, diatoxanthin and diadinoxanthin in diatoms, but these pigments do not usually present as prominent xanthins in the majority of other plants. In contrast to higher plants, diatoms do not biosynthesize luteolin.

However, it was later shown that microalgae, having the diadinoxanthin cycle to produce fucoxanthin for photoprotection, also possess the violaxanthin cycle as found in higher plants [98].

Fucoxanthin, a golden-brown-colored carotenoid pigment, firstly isolated in 1914 [99], is one of the major carotenoids in marine plants. In 1990, its structure was itemized using 2D NMR studies [100]. Fucoxanthin has attracted significant interest as a potential health promoter through its anti-obesity effect as well as its antioxidant, anticancer, anti-inflammatory and other useful properties [101]. Due to the action on the expression of mitochondrial uncoupling protein-1, a key molecule for metabolic thermogenesis, it possesses a fat-burning effect within fat cells in white adipose tissue in rats and mice [102]. Using supplementation with extracts containing fucoxanthin in combination with pomegranate seed oil, it was shown in clinical testing on females with liver diseases that the average weight loss was about 5 kg in obese women over 16 weeks [103]. Fucoxanthin demonstrates anticancer properties, inducing apoptosis of different tumor cells, and its ability to inhibit the invasiveness of cancer cells through the inhibition of expression and secretion of the gelatinolytic enzyme MMP-9 and to suppress the motility of B16-F10 melanoma cells and their adhesion to endothelial cells is particularly important. Moreover, fucoxanthin significantly reduced metastasis in an experimental lung metastasis *in vivo* assay [104]. The diatom *Phaeodactylum tricornutum*, a potential commercial source of fucoxanthin, contains at least ten times more fucoxanthin per gram of dry weight than brown algae [105].

Biosynthesis of fucoxanthin in diatoms is a complicated process, including the formation of β-carotene in the initial stages of the transformation of gerenylgeranyl diphosphate via phytoene, ξ-carotene, and lycopene with the participation of phytoene synthase, phytoene desaturase, and ξ-carotene desaturase. Lycopene forms β-carotene at the action of lycopene β-cyclase. The latter pigment is transformed into zeaxanthin at the catalysis by β-carotene hydroxylase. Further stages include the participation of zeaxanthin epoxidase and lead to antheroxanthin and violaxanthin. All the corresponding genes encoding these enzymes were found in *P. tricornutum*. In addition, many genes encoding violaxanthin de-epoxidase (VDE genes) and zeaxanthin epoxidase (ZEP genes), enzymes participating in the last stages of xanthin biosynthesis in diatoms, were found compared with other phytosynthetic eukaryotes [106,107]. It was suggested that the diversity of VDE and ZEP genes represents early eukaryotic innovations in plants [106].

**Figure 17:**
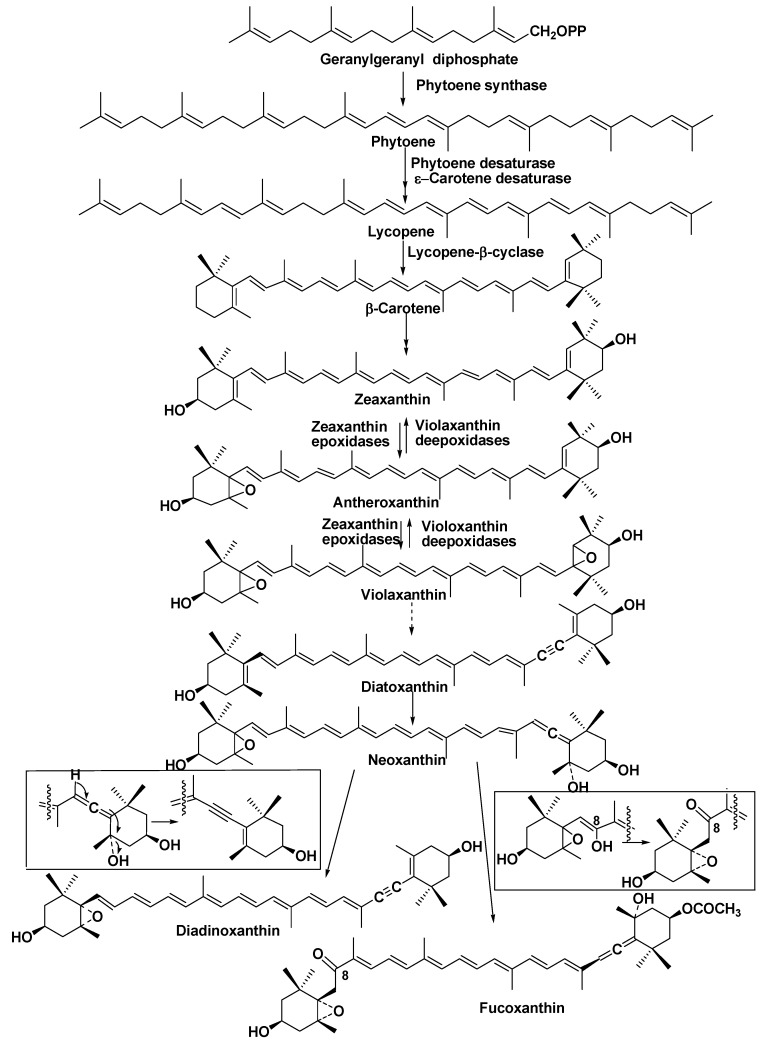
Hypotetical scheme of biosynthesis of fucoxanthin in *P. tricornutum* [108].

Thus, diatoms, which are thought to be derived from secondary symbiosis between autotrophic and heterotrophic eukaryotes, as a consequence of a particular evolutionary history, have an additional xanthophyll-based cycle to dissipate excess light energy. Therefore, several components of carotenoid biosynthesis in diatoms are of ancient origin, and the corresponding enzymes of their biosynthesis were diversified and acquired new functions in these microalgae [107,108]. Neoxanthin, which was isolated from *P. tricornutum*, is a branch point in the biosynthesis of diadinotoxin and fucoxanthin in diatoms. Diadinoxanthin is formed in a single reaction by the formation of an acetylenic bond from an allenic double bond, while the formation of fucoxanthin with acetylation of the hydroxyl group at C3′ proceeds as shown in Figure 17 in the boxes [108].

## 7. Halogenated Compounds

Halogenated methanes such as CHBr_3_, CH_2_Br_2_, CH_2_IBr, CH_3_I and others are produced in oceans by different algae. They play roles as carriers of halogens into the atmosphere and decrease the ozone content in the stratosphere. Laboratory experiments showed that cultures of several marine diatoms also produce halogenated methanes and ethanes. For example, bromoform and dibromomethane were identified in cultures of diatoms belonging to the *Nitzschia* and *Porosira* genera. *Navicula* species possesses an iodoperoxidase, while a bromoperoxidase enzyme was identified in *Nitzschia* sp. [109]. A significant release of hypobromous and hypoiodous acids from polar, temperate and warm water diatoms into their surroundings was also reported. These reactive compounds are equilibrated with molecular halogens (Br_2_ and I_2_) in aqueous solutions [110].

In addition, haloperoxidases in diatoms are involved in diatom-bacteria interactions. A benthic diatom *Nitzschia* cf. *pellucida* induces the H_2_O_2_-dependent inactivation of quorum sensing of bacteria, namely the destruction of *N*-β-ketoacylated homoserine lactones as a result of the cleavage of *N*-acylated chains in these signal molecules [111]. The same marine microalga controls the biofilm formation around themselves, using biogenetic bromine cyanide [112].

**Figure 18:**
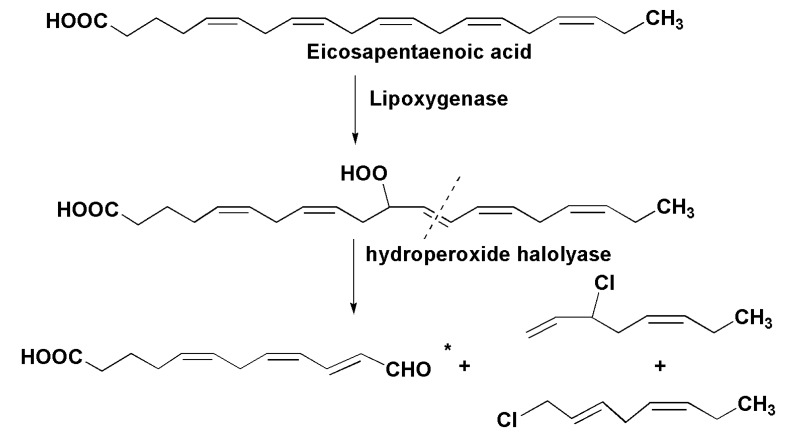
Transformation of eicopentaenic acid into a mixture of chlorinated C8 hydrocarbons in the marine diatom *Stephanopyxis turris* [113]*.*

Some products of lipoxygenase biosynthesis also contain halogen atoms. A transformation of eicosapentaenoic acid into a mixture of chlorinated C8 hydrocarbons is initiated upon disintegration of the marine diatom *Stephanopyxis turris.* Another product of this transformation was identified as 12-oxo acid* (Figure 18). This is the result of the action of a lipoxygenase followed by a cleavage of the intermediate hydroperoxide by a hydroperoxide halolyase [113].

On the whole, halogen-containing metabolites of diatoms and their properties have been less studied than those from other algae, but their harmful influence on other organisms could be significant due to a great contribution of diatoms in biomass of marine biota.

## 8. Toxins

**Figure 19:**
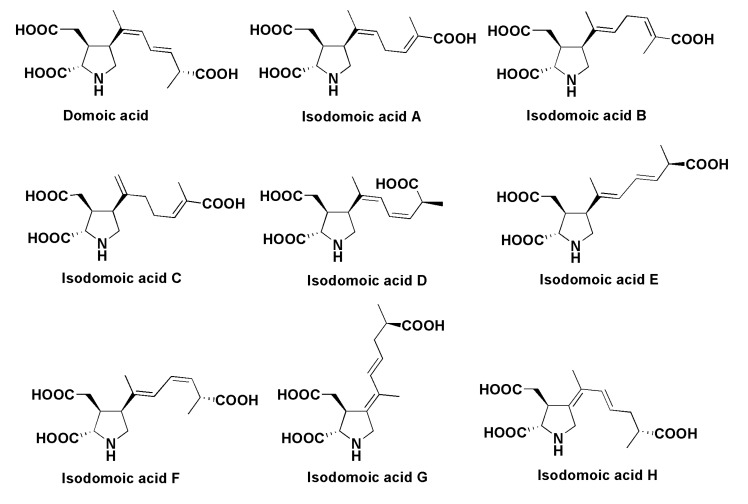
Structures of domoic and isodomoic acids.

Domoic acid (DA) (Figure 19), a product of mixed biogenesis from amino acids and an isoprenoid precursor, is a toxin of diatoms, which is accumulated in mollusks and other marine animals and causes amnesic shellfish poisoning (ASP) or domoic acid poisoning (DAP). The first case of this type of poisoning, caused by eating cultivated mussel *Mytilus edulis*, was described in Canada in 1987. In that case four people died and over 100 people had various toxic syndromes, including an 84-year-old male, who had symptoms of temporal epilepsy, caused by this intoxication [114,115,116]. Domoic acid was originally isolated from the red alga *Chondria armata* [117], and its structure was later revised after chemical synthesis [118]. This alga is known as “domoi” in Japan. After the discovery of ASP it was found that the toxin is also produced by some diatoms such as *Pseudo-nitzschia pungens*, *Pseudo-nitzschia* spp. and *Nitzschia navis-virginica* [119,120,121]. Japanese scientists showed that the diatom *Pseudo-nitzshia multiseries* partly loses the ability to produce DA when cultivated axenically, but recovers this ability when marine bacteria from the original culture are added to axenic cultures [122]. This means that production requires the joint participation of microalgae and bacteria. The origin of domoic acid in diatoms as well as the unexpected biochemical parallelism in the presence of the toxin in both the red algae *Chondria armata* and diatoms continue to be intriguing sealed books.

The toxic action of domoic acid is characterized by a series of clinical symptoms, including brain pathology with cell/tissue injury and memory impairment [123,124]. After the amnesic shellfish poisoning incident in Quebec in 1987 [114,115,116], DA was found in seafood by different methods [125] from different geographic areas [126,127,128] and numerous DAP cases occurred, partly as a consequence of chronic sub-lethal exposure to the toxin [129]. For example, 400 sea lions were found onshore in California in 1988, and their poisoning was correlated with a bloom of the diatom *Pseudo-nitzschia australis*, when domoic acid contaminated anchovies [129]. Domoic epileptic disease is accompanied by spontaneous recurrent seizures over several weeks to months, and this disease needs improved medical diagnosis, treatment and prognosis.

Several isomers of DA, so-called isodomoic acids, (Figure 19) were isolated from the alga *Chondria armata* [130] and later they were identified in mussels contaminated by microalgae [131]. It was shown that DA may be transformed into isodomoic acids following irradiation by sunlight. The chemical properties of domoic and isodomoic acids were also studied [132].

Domoic acid and its producers are widely distributed in the world’s oceans, and the increasing frequency of toxic *Pseudo-nitzschia* algal blooms seems to be, at least partly, attributable to human activity [133]. The blooms usually occur at a time when the seawater temperature is falling, at low light and changes in salinity [134]. In the Western North Pacific, 314 phytoplankton samples collected from 1995 to 2006 were studied and the presence of 11 *Pseudo-nitzschia* species was revealed [135]. In studies on 35 stations from the Pacific subarctic to the Southern Ocean, *Pseudo-nitzschia* were detected not only in coastal waters, but in near-surface oceanic waters with levels of domoic acid from 0.3 fg to 2 pg per cell. In the Antarctic Pacific, DA reached 220 ng per L, a level at which sea animal mortalities take place. It was concluded that this neurotoxin occurs both in oceanic and coastal waters, and the variation in its amounts is connected with changes in *Pseudo-nitzschia* abundance, climate cycles and artificial iron fertilization [136].

## 9. Miscellaneous Natural Products

### 9.1. Attractants

**Figure 20:**
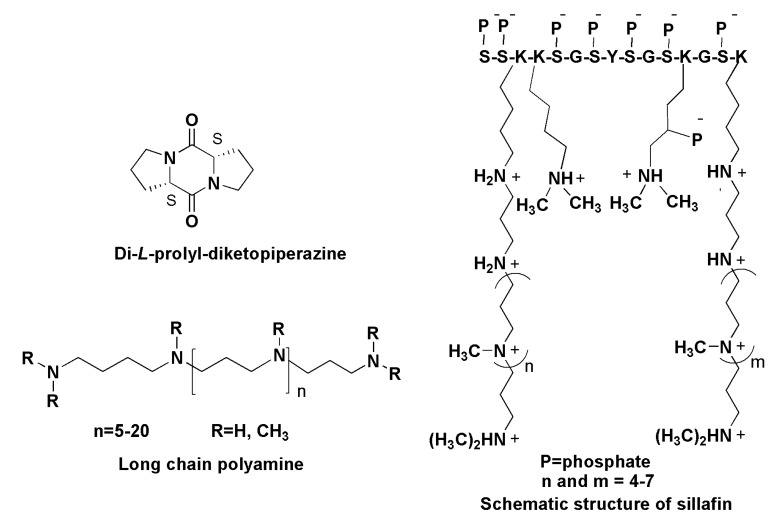
Some natural products of other chemical natures.

Some diatoms induce other chemical signals to attract sexual partners. This was confirmed by using the metabolomic approach in the comparison of the metabolic profiles of sexually active and inactive forms of the unicellular diatom *Seminavis robusta*. The attracting mating form (MT^−^cells) generated the pheromone identified as di-l-prolyl-diketopiperazine, causing the migratory behavior of MT cells [137,138]. Chiral separation of diketopiperazines from the medium, in which the diatom was cultivated was carried out using supercritical fluid chromatography combined with ESI MS and gave, besides the dominant l,l-isomer (Figure 20), only trace amounts of di-d-prolyl-diketopiperazine [139].

### 9.2. Long-Chain Polyamines

Silica biomineralization dominates in such biological forms as diatoms, radiolaria, synurophytes and sponges, and as a consequence silica is the second most abundant biomineral in nature after biogenic CaCO_3_. Diatoms as biomineralizing organisms contain species-specific biochemical complexes in their silica deposition vesicles to produce biosilica for ornate cell walls. The biosilica is composed of amorphous, hydrated SiO_2_ and contains organic macromolecules. Each diatom is equipped with a special set of silica-precipitated proteins, known as silaffins, and long-chain polyamines (LCPAs) [140,141,142].

Silaffins, phosphoproteins enriched by Ser, Thr and Lys have covalently modified lysine-lysine elements bearing polyamines consisting of six and more repeats of the amonopropylene units. Silaffins were suggested to be inducers and templates for silica deposition. Isolated from the cell walls of diatoms they provide, together with LCPAs, the generation of networks of silica nanospheres within seconds after addition to silicic acid solution delivered into the cells by silicon transporter proteins [143,144]. The silaffin-dependent mechanism includes the interaction of silaffins with LCPAs inside the silica deposition vesicles and gives a particular biosilica material with regularly located pores of different sizes and forms in each species. This natural species-specific fabrication allows taxonomists to identify thousands of diatom species. Long-chain polyamines (LCPAs, Figure 20) with molecular masses ranging between 600 and 1500 Da, belonging to secondary metabolites, are characterized by unique compositions in each species studied and may be found both free and bound to silaffins’ or related proteins forms. These compounds, usually based on putrescine with attached aminopropylene units, are major organic constituents of biosilica and the longest polyamine chains found in nature (sometimes more than 20 repeated units). They effectively precipitate biosilica with the formation of nanospheres. Using electrospray ionization mass spectrometric analysis, it was established that the polyamines from the diatom *Cylindrotheca fusiformis* form the simplest composition, comprising only six molecular species. Polyamines differ from each other mainly in molecular masses and methylation level, in some cases not only terminal, but also internal quaternary *N*-atoms present in LCPAs [145,146].

## 10. Genomic and Post-Genomics Approaches to the Studies on Bioactive Low-Molecular Metabolites from Diatoms

In recent years a series of papers directed at the sequencing of nuclear, mitochondrial or chloroplast genomes of diatom microalgae were published [19,147,148,149,150]. Although application of genomic and post-genomic approaches to the studies on low-molecular-weight metabolites from diatoms in itself did not result in increasing the number of known compounds or in the discovery of their new biological activities, the corresponding works are very important in the respect of the understanding of the biosyntheses and the opening up of new biotechnological prospects for the practical use of these metabolites. Herein, we give several examples of these types of studies.

Genes, encoding lipid-biosynthesis enzymes, which are of interest due to the potential of diatoms as a renewable source for biodiesel and the importance of ω-3 polyunsaturated fatty acids for human health were found in the genomes of several diatoms [151]. For instance, in the genome of the diatom *Thalassiosira pseudonana*, DNA sequences, encoding a long-chain polyunsaturated acyl-coenzyme A synthetase were identified. The yeast *Saccharamyces cerevisiae,* transformed with the algal gene was able to incorporate more health-beneficial PUFA in triacylglycerols in comparison with control yeast [152]. In the recent review on the application of genetic engineering methods to cultivated diatoms as a way to a new alternative source of PUFA, several impressive experimental results were discussed. Detailed pathways of PUFA biosynthesis in diatoms was proposed on the basis of nucleotide sequences in the genomes of *T. pseudonana* and *P. tricornutum*, encoding mainly different desaturases [153]. The application of genetic engineering techniques and the selection of conditions for overexpressing the corresponding enzymes led to a significant increase in the proportion of eicosapentaenoic acid and in algal lipid production of the diatom *Phaeodactylum tricornutum* [154,155].

It is of particular interest that after the introduction of a gene, encoding a glucose transporter, transgenic *P. tricornutum* becomes heterotrophic and capable of growing without light [156]. This achievement shows that introducing even one gene can lead to pervasive changes in the biochemistry and physiology of diatoms.

Polyamines are another important group having possible application, particularly in the field of the potential use of biosilica in nanoelectronics. Recently, analysis of genomes of *T. pseudonana* and *P. tricornutum* revealed molecular machines, which dimethylate and transfer multiple aminopropyl unit polyamines onto sillafin proteins [157].

Identification of candidate genes, encoding enzymes of the carotenoid biosynthesis in the diatom *P. tricornutum* has allowed the proposal of the hypothetical pathways from both the 2-C-methyl-d-erythritol 4-phosphate and dimethylallyl and isopentenyl diphosphates to fucoxanthin [158].

Using gene silencing and heterologous gene expression approaches as well as enzyme inhibitors, new data on sterol biosynthesis in *P. tricornutum* were obtained [159].

As mentioned above, the capability of diatoms belonging to the *Pseudo-nitzschia* genus to produce amnesic toxin (domoic acid) is an intriguing peculiarity of these microalgae. Through microarray analysis of the cultures of diatom *P. multiseries* with high- and low-domoic acid productions, a new approach to the understanding of genes involved in the biosynthesis of domoic acid was proposed [160].

Armbrust *et al.* [161] reported nucleotide sequences of the 34 million-base pair nuclear genome, 129 thousand-base pair plastid and 44 thousand-base pair mitochondrial genomes of the marine diatom *Thalassiosira pseudonana*. New genes for silica transport and formation of silica cell walls as well as genes encoding polyunsaturated fatty acid biosynthesis along with other genes allowing diatoms to prosper in marine environment were identified. A comparative analysis of genes involved in carbon partitioning metabolic pathways in *T. pseudonana*, *P. tricornutum* and *Fragilariopsis cylindricus* [162] showed substantial differences between analysed species and revealed that the diatoms may regulate the carbon flux to adapt to environmental niches.

Thus, in their majority, genes encoding enzymes of lipid and carotenoid biosyntheses or those connected with biosilification were so far found in genomes of diatoms. However genomic and post-genomic approaches might contribute to detecting the diatom species, perspective for the isolation of new small molecules in the case of finding the genes encoding enzymes, usually participating in biosynthesis of low-molecular-weight metabolites, for example such as polyketide and other synthases.

## 11. Conclusions

Diatoms are a dominant and important group of eukaryotic microalgae, inhabiting all aqueous environments during the last near 200 millions years. In oceans, they produce a tremendous biomass, making up more than 20% of the primary biological production on earth and funneling biomass and energy to higher levels [1]. Diatoms represent the basis of the trophic food web in marine biota and are used as food for mariculture. Herein, the various natural products of diatoms, from polyunsaturated fatty acids to heterocyclic toxins, were reviewed in accordance with their chemical structural groups. The majority of the studies concerned the lipids from diatoms and in particular oxylipins. The corresponding biosynthetic pathways liberate polyunsaturated fatty acids from glyco- and other lipids, and give rise (through lipoxygenase/hydroperoxide lyase and other enzymatic systems) to numerous ecologically important chemical groups, including unsaturated aldehydes, fatty acid epoxy alcohols and pheromones. Besides regulation of species-species relations, low-molecular-weight metabolites of diatoms participate in other globally important processes such as influencing the ozone level in the stratosphere and biomineralization. Some diatom metabolites are used in studies on the climatic changes that have taken place during the last geologic periods. However, it should be mentioned that the diversity of the low molecular weight natural products of these organisms has not as yet been sufficiently studied and further investigations should promise new and interesting findings.

## References

[1] Mann D.G. (1996). Biodiversity, biogeography and conservation of diatoms. Hydrobiologia.

[2] Mann D.G. (1989). The species concept in diatoms: Evidences for morphologically distinct, sympatric gamodemes in four epipelic species. Plant Syst. Evol..

[3] Round F.E., Crawford D.G., Mann D.G. (1990). The Diatoms: Biology and Morphology of the Genera.

[4] Pivateau F., Gandemer G., Baud J.-P. (1999). Changes in lipid and fatty acids compositions of European oysters fattened with *Skeletonema costatum* diatom for six weeks in ponds. Aquac. Int..

[5] Ackman R.G., Jangaard P.M., Hoyle R.J., Brosherholf H. (1964). Origin of marine fatty acids. I. Analysis of fatty acids produced by the diatom *Skeletonema costatum*. J. Fish. Res. Board Can..

[6] Ackman R.G., Tocher C.S., McLahlan J. (1968). Marine phytoplankter fatty acids. J. Fish. Res. Board Can..

[7] Brokerhoff H., Yurkowsky M., Hoyle R.J., Ackman R.G. (1964). Fatty acids distribution in lipids of marine phytoplankton. J. Fish. Res. Board Can..

[8] Ackman R.G. (2003). A history of fats and oils in Canada. Lipids.

[9] Dunstan G.A., Volkman J.K., Barret S.M., Leroi J.-M., Jeffrey S.W. (1994). Essential polyunsaturated fatty acids from 14 species of diatom (Bacillariophyceae). Phytochemistry.

[10] Viso A.C., Marty J.C. (1993). Fatty acids from 28 marine microalgae. Phytochemistry.

[11] Viron C., Saunois A., Andre P., Perly B., Lafosse M. (2000). Isolation and identification of unsaturated fatty acid methyl esters from marine microalgae. Anal. Chim. Acta.

[12] Arao T., Kawaguchi A., Yamada M. (1987). Positional distribution of fatty acids in lipids of the marine diatom *Phaeodactylum tricornutum*. Phytochemistry.

[13] Medina A.R., Grima E.M., Gimenez A.G., Gonzales M.J.I. (1998). Downstream processing of algal polyunsaturated fatty acids. Biotechnol. Adv..

[14] Vereshchagin A.L., Glyzina O.Y., Basharina T.N., Safonova T.A., Latyshev N.A., Liubochko S.A., Korneva E.S., Petrova D.P., Annenkov W., Danolovtseva E.N. (2008). Culturing of a fresh-water diatom alga *Synedra acus* in a 100L bioreactor and analysis of the biomass composition. Biotekhnologia.

[15] Shishlyannikov S.M., Klimenkov I.V., Bedoshvili E.D., Mikhailov I.S., Gorshkov A.G. (2014). Effect of mixotrophic growth on the ultrastructure and fatty acid composition of the diatom *Synedra acus* from Lake Baikal. J. Biol. Res.–Thessalon..

[16] Arao T., Yamada M. (1993). Biosynthesis of polyunsaturated fatty acids in the marine diatom *Phaeodactylum tricornutum*. Phytochemistry.

[17] Mansour M.P., Frampton D.M.F., Nichols P.D., Volkman J.K., Blackburn S.I. (2005). Lipid and fatty acid yield of nine stationary-phase microalgae, applications and unusual C24–C28 polyunsaturated fatty acids. J. Appl. Phycol..

[18] Arao T., Yamada M. (1994). Biosynthesis of polyunsaturated fatty acids in the marine diatom *Phaeodactylum tricornotum*. Phytochemistry.

[19] Bowler C., Allen A.E., Badger J.H., Grimwood J., Jabbari K., Kuo A., Maheswari U., Martens C., Maumas F., Otillar R.P. (2008). The *Phaeodactylum* genome reveals the evolutionary history of diatom genomes. Nature.

[20] Tanaka T., Fukuda Y., Yoshino T., Maeda Y., Muto M., Matsumoto M., Mayama S., Matsunaga T. (2011). High throughput pyrosequencing of the chloroplast genome of a highly neutral-lipid-producing marine pennate diatom, *Fistulifera* sp., strain JPCC DA 0580. Photosynth. Res..

[21] Nojima D., Yoshino T., Maeda Y., Tanaka M., Nemoto M., Tanaka T. (2013). Proteomics analysis of oil body-associated proteins in the oleaginous diatom. J. Proteome Res..

[22] Liang Y., Maeda Y., Sunaga Y., Muto M., Matsumoto M., Yoshino T., Tanaka T. (2013). Biosynthesis of polyunsaturated fatty acids in the oleaginous marine diatom *Fistulifera* sp. Strain JPCC DA 0580. Mar. Drugs.

[23] Berge J.P., Couygou J.P., Dubacq J.P., Durand P. (1995). Reassessment of the lipid composition of the diatom, *Skeletonema costatum*. Phytochemistry.

[24] Hildebrand M., Davis A.K., Smith S.R., Trailer J.C., Abbriano R. (2012). The place of diatoms in the biofuel industry. Biofuels.

[25] Sharma K.K., Schuhmann H., Schenk P.M. (2012). High lipid induction in microalgae for biodiesal production. Energies.

[26] Thompson G.A. (1996). Lipid and membrane function in green algae. Biochim. Biophys. Acta.

[27] Yu E.T., Zendejas E.J., Lane P.D., Gaucher S., Simmons B.A., Lane T.W. (2009). Triacylglycerols accumulation and profiling in the model diatoms *Thalassiosira pseudonana*, and *Phaeodactylum tricornutum* (Baccilarriophyceae) during starvation. J. Appl. Phycol..

[28] Siron R., Giusti’ G., Berland B. (1989). Changes in the fatty acid composition of *Phaeodactylum tricornutum* and *Dunaliella tertiolecta* during growth and under phosphorus deficiency. Mar. Ecol. Prog. Ser..

[29] Li S., Xu J.L., Chen J., Chen J.J., Zhou C.X., Yan X.J. (2014). Characterization of the triacylglycerol profile in marine diatoms by ultra performance liquid chromatography coupled with electrospray ionization Q1-quadrupole time-of-flight mass spectrometry. J. Appl. Phycol..

[30] Roughan P.G., Slack C.R. (1984). Glycerolipid synthesis in leaves. Trends Biochem. Sci..

[31] Hu Q., Sommerfeld M., Jarvis E., Ghirardi M., Posewitz M., Seibert M., Darzins A. (2008). Microalgal triacyglycerols as feedstocks for biofuel production, perspectives and advances. Plant J..

[32] Yao Y., Peng K.T., Huang T., Niu Y.F., Xie W.H., Yang W.D., Liu J.S., Li H.Y. (2014). Glycerol and neutral lipid production in the oleaginous marine diatom promoted by overexpression of glycerol-3-phosphate dehydrogenase. Biotechnol. Biofuels.

[33] Liang Y., Maeda Y., Yoahino T., Matsumoto M., Tanaka T. (2014). Profiling of polar lipids in marine oleagineous diatom *Fistulifera* sp. JPCC DA0580: Prediction of the potential mechanism for eicosapentaenic acid-incorporation into triacylglycerols. Mar. Drugs.

[34] Anderson R., Kates M., Volcani B.E. (1978). Identification of the sulfolipids in the non-synthetic diatom *Nitzschia alba*. Biochem. Biophys. Acta.

[35] Bissert P., Ito S., Tremblay P.A., Volcani B.E., Dessort D., Kates M. (1984). Occurrence of phosphatidylsulfocholine, the sulfonium analog of phospatidylcholine in some diatoms and algae. Biochim. Bophys. Acta.

[36] Anderson R., Kates M., Volcani B.E. (1979). Studies on the biosynthesis of sulfolipids in the diatoms. Biochem. Biophys. Acta.

[37] Kabara J.J., Swieczkowsky D.M., Conley A.J., Truant J.P. (1972). Fatty acids and derivatives as antimicrobial agents. Antimicrob. Agents Chemother..

[38] Jüttner F. (2001). Liberation of 5,8,11,14,17-eicosapentaenic acid and other polyunsaturated fatty acids as a grazer defense reaction in epilithic diatom biofilms. J. Phycol..

[39] Sieg R.D., Poulson-Ellestad K.L., Kubanek J. (2011). Chemical ecology of the marine plankton. J. Nat. Prod. Rep..

[40] Poulson K.L., Sieg R.D., Kubanek J. (2009). Chemical ecology of the marine plankton. Nat. Prod. Rep..

[41] Paul V.L., Ritson-Williams R. (2008). Marine Chemical Ecology. Nat. Prod. Rep..

[42] Miralto A., Barone G., Romano G., Poulet S.A., Ianora A., Russo G.L., Buttino I., Mazzarella G., Laabir M., Cabrini M. (1999). The insidious effect of diatoms on copepod reproduction. Nature.

[43] Irigoien X., Harris R.P., Verheye H.M., Joly P., Runge J., Starr M., Pond D., Campbell R., Shreeve R., Ward P. (2002). Copepod hatching success in marine ecosystems with high diatom concentrations. Nature.

[44] Bartual A., Arandia-Gorostidi N., Cozar A., Morillo-Garcia S., Ortega M.J., Vidal M., Cabello A.M., Gonzales-Cordillo J.I., Echevarria F. (2014). Polyunsaturated aldehydes from large phytoplankton of the Atlantic Ocean Surface (42° N to 33° S). Mar. Drugs.

[45] Vardi A., Formiggini F., Casotti R., de Martino A., Ribalet F., Miralto A., Bowler C. (2006). A stress surveillance system based on calcium and nitric oxide in marine diatoms. PLoS Biol..

[46] Lauritiano C., Borra M., Carotenuto Y., Biffali E., Mralto A., Procaccini G., Ianora A. (2011). First molecular evidence of diatom effects in the copepod *Calanus elgolandicus*. J. Exp. Mar. Biol. Ecol..

[47] Ianora A., Miralto A., Poulet S.A., Carotenuto Y., Buttino I., Romano G., Casotti R., Ponhert G., Wichard T., Colucci-D’Amato L. (2004). Aldehyde suppression of copepod recruitment in blooms of a ubiquitous planktonic diatom. Nature.

[48] Wichard T., Poulet S.A., Halsband-Lenk C., Albana A., Harris R., Liu D., Pohnert G. (2005). Studies on the chemical defense potential of diatoms: Screening of fifty one species for alpha, beta, gamma, delta-unsaturated aldehydes. J. Chem. Ecol..

[49] Fontano A., D’Ippolito G., Cutignano A., Miralto A., Ianora A., Romano G., Cimino G. (2007). Chemistry of oxylipin pathways in marine diatoms. Pure Appl. Chem..

[50] D’Ippolito G., Romano G., Caruso T., Spinella A., Cimino G., Fontano A. (2003). Production of octadienal in the marine diatom *Skeletonema costatum*. Org. Lett..

[51] D’Ippolito G., Romano G., Iadicicco O., Miralto A., Ianara A., Cimino G., Fontana A. (2002). New birth-control aldehydes from the marine diatom *Skeletonema costatum*: Characterization and biogenesis. Tetrahedron Lett..

[52] D’Ippolito G., Citignano A., Briante R., Febbrario R., Cimino G., Fontana A. (2005). New C16 fatty-acid based oxylipin pathway in the marine diatom *Thalassiosira rotula*. Org. Biomol. Chem..

[53] Barriero A., Carotenuto Y., Lamari N., Esposito F., D’Ippolito G., Fontana A., Romano G., Ianora A., Miralto A., Guisande C. (2011). Diatom induction of reproductive failure in copepod: The effect of PUAs *versus* non volatile oxylipins. J. Exp. Mar. Biol. Ecol..

[54] D’Ippolito G., Lamari N., Montresor M., Romano G., Cutignano A., Gerecht A., Cimino G., Fontana A. (2009). 15S-lipogenase metabolism in the marine diatom *Pseudo-nitzschia dilicatissima*. New Phytol..

[55] Nanjappa D., D’Ippolito G., Gallo C., Zingone A., Fontana A. (2014). Oxylipin diversity in the diatom family Leptocylindraceae reveals DHA derivatives in marine diatoms. Mar. Drugs.

[56] Wang R., Shimizu Y. (1990). Bacillariolides I and II, a new type of cyclopentane eicosanoids from the diatom *Nitzschia pungens*. J. Chem. Soc. Chem. Commun..

[57] Zheng N., Shimizu Y. (1997). The isolation and structure of bacillariolide III, an extracellular metabolite of the diatom *Pseudo-nitschia multiseries*. J. Chem. Soc. Chem. Commun..

[58] Wang R., Shimizu Y., Steiner J.R., Clardy J.J. (1993). The absolute configuration of bacillariolides I and II, a new type of cyclopentane eicosanoids from a marine diatom. Chem. Soc. Chem. Commun..

[59] Jüttner F., Müller H. (1979). Excretion of octadiene and octatrienes by a freshwater diatom. Naturwissenschaften.

[60] Derenbach J.B., Pesando D. (1986). Investigations into a small fraction of volatile hydrocarbons. III. Two diatom cultures produce ectocarpene, a pheromone of brown algae. Mar. Chem..

[61] Wendel T., Jüttner F. (1996). Lipoxygenase-mediated formation of hydrocarbons and unsaturated aldehydes in freshwater diatoms. Phytochemistry.

[62] Pohnert G., Boland W., Pohnert G., Boland W. (1996). Biosynthesis of the algal pheromone hormosirene by the freshwater diatom *Gomphonema parvulum* (Bacillariophyceae). Tetrahedron.

[63] Hombeck M., Boland W. (1998). Biosynthesis of the algal pheromone fucoserratene by the freshwater diatom *Asterionella formosa* (Bacillariophyceae). Tetrahedron.

[64] Volkman J.K. (1986). A review of sterol marker of for marine and terrigenous organic matter. Org. Geochem..

[65] Volkman J.K., Barrett S.M., Dunstan G.A., Jeffrey S.W. (1993). Geochemical significance of the occurrence of dinosterol and other 4-methyl sterols in a marine diatom. Org. Geochem..

[66] Rampen S.W., Abbas B.A., Schouten S., Damste J.J.S. (2010). A comprehensive study of sterols in marine diatoms (Bacillariophyta): Implication of their use as tracers for diatom productivity. Limnol. Oceanogr..

[67] Volkman J.K., Hallegraeff G.M. (1988). Lipid in marine diatoms of the genus *Thalassiosira*: Predominance of 24-methylenecholesterol. Phytochemistry.

[68] Ponomarenko L.P., Stonik I.V., Aizdaicher N.A., Orlova T.J., Popovskaya G.I., Pomazkina G., Stonik V.A. (2004). Sterols of marine microalgae *Pyramimonas* cf. *cordata* (Prasinophyta), *Atthea ussirensis* sp. nov. (Bacillariophyta) and a spring diatom bloom from Lake Baikal. Comp. Biochem. Physiol..

[69] Volkman J.K., Barrett S.M., Blackburn S.I., Mansour M.P., Sikes E.L., Gelin F. (1998). Microalgal biomarkers: A review of recent research developments. Org. Geochem..

[70] Kalinovsky A.I., Gorshkov A.G., Ponomarenko L.P., Stonik V.A., Dmitrenok A.S., Grachev M.A. (2010). Preparation of 13 C-24-methylcholesta-5,24(28)-dien-3β-ol by cultivation of the Baikal diatom *Synedra acus* in NaH^13^CO_3_. Russ. Chem. Bull..

[71] Ballantine J.A., Lavis A., Morris R.J. (1979). Sterols of phytoplankton: Effects of illumination and growth stage. Phytochemistry.

[72] Masse G., Belt S.T., Rowland S.J., Rohmer R. (2004). Isoprenoid biosynthesis in the diatoms *Rhisosolenia setigera* (Brightwell) and *Haslea osrearia* (Simonsen). Proc. Natl. Acad. Sci. USA.

[73] Blumer M., Mullin M.M., Guillard R.R.L. (1970). A polyunsaturated hydrocarbon (3,6,9,12,15,18-heneicosahexaene) in the marine food web. Mar. Biol..

[74] Lee R.F., Loeblich A.R. (1971). Distribution of 21:6 hydrocarbon and its relationship to 22:6 fatty acid in algae. Phytochemistry.

[75] Volkman J.K., Barrett S.M., Dunstan G.A. (1994). C_25_ and C_30_ highly branched alkenes in laboratory cultures of two marine diatoms. Org. Geochem..

[76] Grossi V., Beker B., Geenevasen J.A.J., Schouten S., Raphel D., Fontaine M.-F., Damste J.S.S. (2004). C_25_-highly branched isoprenoid alkenes from the benthic diatom *Pleurosigma strigosum*. Phytochemistry..

[77] Damste J.S.S., Schouten S., Rijpsta W.I.C., Hopmans E.C., Peletier H., Gieskes W.W.C., Geenevasen J.A.J. (2000). Novel polyunsaturated *n*-alkenes in the marine diatom *Rhizosolenia setigera*. Eur. J. Biochem..

[78] Kardys M. (1980). Uptake of hydrocabons by the marine diatom *Cyclotella cryptica*. Micrbial Ecol..

[79] Robson J.N., Rowland S.J. (1986). Identification of novel widely distributed sedimentary acyclic sesterterpenoids. Nature.

[80] Robson J.N., Rowland S. (1988). Synthesis of highly branched C_30_ sedimentary hydrocarbon. Tetrahedron Lett..

[81] Belt S.T., Cooke D.A., Robert J.M., Rowland S. (1996). Structural characterization of widespread polyunsaturated isoprenoid biomarkers: A C_25_ triene, tetraene, and pentaene from the diatom *Haslea ostrearia* Simonsen. Tetrahedron Lett..

[82] Wraige E.J., Belt S.T., Lewis C.A., Cooke D.A., Robert J.-M., Masse G., Rowland S.J. (1997). Variations in structures and distributions of C_25_ highly branched isoprenoids in cultures of the diatom, *Haslea ostrearia* (Simonsen). Org. Geochem..

[83] Wraige E.J., Johns L., Belt S.T., Masse G., Robert J.-M., Rowland S.J. (1999). Highly branched C-25 isoprenoids in axenic cultures of *Haslea ostrearia*. Phytochemistry.

[84] Sininqhe Damste J.S., Schouten S., Rijpsta W.I.C., Hopmans E.C., Peletier H., van der Maarel M.J.E.C., Gieskes W.W.C., Geenwasen J.A.J. (1999). Structural identification of the C25 highly branched pentaene in the marine diatom, *Rhizosolenia setigera*. Org. Geochem..

[85] Damste J.S.S., Rijpsta W.I.C., Schouten S., Peletier H., van der Maarel M.J.E.C., Gieskes W.W.C. (1999). A C_25_ highly branched isoprenoid alkene and C25 and C27 *n*-polyenes in the marine diatom, *Rhizosolenia setigera*. Org. Geochem..

[86] Rowland S.J., Allard W.G., Belt S.T., Masse G., Robert J.-M., Blackburn S., Frampton D., Revill A.T., Volkman J.K. (2001). Factors influencing the distribution of polyunsaturated terpenoids in the diatom *Rhizololenia setigera*. Phytochemistry.

[87] Belt S.T., Allard W.G., Masse G., Robert J.-M., Rowland S.R. (2000). Highly branched isoprenoids (HBIs): Identification of the most common and abundant sedimentary isomers. Geochim. Cosmochim. Acta.

[88] Belt S.T., Masse G., Allard W.G., Robert J.-M., Rowland S.J. (2001). C25 highly branched isoprenoid alkenes in planktonic diatoms of the *Pleurosigma* genus. Org. Geochem..

[89] Nichols P.D., Palmisano A.C., Smith A.G., White D.C. (1986). Lipids from the Antharctic sea ice diatom *Nitzschia cylindrus*. Phytochemistry.

[90] Nichols P.D., Volkman J.K., Palmisano A.C., Smith A.G., White D.C. (1988). Occurrence of an isoprenoid C_25_ diunsaturated alkene and high neutral lipid content in Antarctic sea-ice diatom communities. J. Phycol..

[91] Nichols D.S., Nichols P.D., Sullivan C.W. (1993). Fatty acid, sterols and hydrocabon composition of Antarctic sea ice diatom communities during the spring bloom in McMurdo sound. Antarct. Sci..

[92] Belt S.T., Masse G., Poulin M., Michel C., LeBlank B. (2007). A novel chemical fossil of palaeosea ice: IP_25_. Org. Geochem..

[93] Brown T.A., Belt S.T., Tatarek A., Mundy C.J. (2014). Source identification of the Arctic sea ice proxy IP_25_. Nat. Commun..

[94] Belt S.T., Allard W.G., Masse G., Robert J.M., Rowland S.J. (2001). Structural characterization of C_30_ highly branched isoprenoid alkenes (rhizenes) in the marine diatom *Rhizosolenia setigera*. Tetrahedron Lett..

[95] Belt S.M., Masse G., Allard W.G., Robert J.M., Rowland S.J. (2003). Novel monocyclic sester- and triterpenoids from the marine diatom, *Rhizosolenia setigera*. Tetrahedron Lett..

[96] Pennington F., Guillard R.R.L., Jensen S. (1988). Carotenoid distribution patterns in Bacillariophyceae (diatoms). Biochem. Syst. Ecol..

[97] Zapata M., Rodriguez F., Fraga S., Barra L., Ruggiero M.V. (2011). Chlorophyll c pigment patterns in 18 species (51 strains) of the genus *Pseudo-nitzschia* (Bacillariophyceae). J. Phycol..

[98] Lohr M., Wilhelm C. (1999). Algae displaying the diadinoxanthin cycle also possess the violaxanthin cycle. Proc. Natl. Acad. Sci..

[99] Willstätter R., Page H.R. (1914). Chlorophyll. XXIV. The pigments of the brown algae. Justus Liebigs Ann. Chem..

[100] Englert G., Bjornland T., Liannen-Jensen S. (1990). 1D and 2D NMR study of some allenic carotenoids of the fucoxanthin series. Magn. Reson. Chem..

[101] Wang J.-H., Wu C.-F., Yuan J.-P., Yuan P.J. (2011). Fucoxanthin, a marine carotenoid present in brown seaweeds and diatoms: Metabolism and bioactivities relevant to human health. Mar. Drugs.

[102] Maeda H., Hosokawa M., Sashima T., Funayama K., Miyashita K. (2005). Fucoxanthin from edible seaweed, *Undaria pinnatifida*, shows antiobesity effect through UCP-1 in white adipose tissues. Biochem. Biophys. Res. Commun..

[103] Abidov M., Ramazanov Z., Seifulla R., Grachev S. (2010). The effect of Xanthigen in the weight management of obese premenopausal women with non-alcoholic fatty liver disease and normal liver fat. Diabetes Obes. Metab..

[104] Chung T.W., Choi H.J., Lee J.Y., Jeong H.S., Kim C.H., Joo M., Choi J.Y., Han S.W., Kim S.Y., Choi J.S. (2013). Marine algal fucoxanthin inhibits the metastatic potential of cancer cells. Biochem. Biophys. Res. Commun..

[105] Kim S.M., Yung Y.I., Kwon O.-N., Cha K.H., Um B.H., Chung D., Pan C.-H. (2012). A potential commercial source of fucoxanthin extracted from the microalga *Phaeodactylum tricornutum*. Appl. Biochem. Biotechnol..

[106] Lohr M., Wilhelm C. (2001). Xanthophyll synthesis in diatoms: Quantification of putative intermediates and comparison of pigment conversation kinetics with rate constants derived from a model. Planta.

[107] Coesel S., Obornik M., Varela J., Falciotore A., Bowler C. (2008). Evolutionary origin of the carotenoid biosynthetic pathway in marine diatoms. PLoS ONE.

[108] Dambek M., Eilers U., Breitenbach J., Steiger S., Büchel C., Sandmann G. (2012). Biosynthesis of fucoxanthin and diadinoxan*t*hin and function of initial pathways genes in *Phaeodactylum tricornutum*. J. Exp. Bot..

[109] Moore R.M., Webb M., Tokarczuk R., Wever R.J. (2012). Bromoperoxidase and iodoperoxidase enzymes and production of halogenated methanes in marine diatom cultures. J. Geophys. Res..

[110] Hill V.L., Manley S.L. (2009). Release of reactive bromine and iodine from diatoms and its possible role in halogen transfer in polar and tropical oceans. Limnol. Oceanogr..

[111] Syrpas M., Ruysbergh E., Blommaert L., Vanelslander B.M., Sabbe K., Vyverman W., de Kimpe N., Mangelinckx S. (2014). Haloperoxidase mediated quorum quenching by *Nitzschia* cf. *pellucida*: Study of the metabolization of *N*-acyl homoserine lactones by a bentic diatom. Mar. Drugs.

[112] Bart V., Carsten P., Grueneberg J., Prince E.K., Gillard J., Sabbe K., Pohnert G., Vyverman W. (2012). Daily bursts of biogenic cyanogen bromide (BrCN) controls biofilm formation around a marine benthic diatom. Proc. Natl. Acad. Sci. USA.

[113] Wichard T., Pohnert G. (2006). Formation of halogenated medium chain hydrocarbons by a lipoxygenase/hydroperoxide halolyase-mediated transformation in planktonic microalgae. J. Am. Chem. Soc..

[114] Quilliam M.A., Wright J.L. (1989). The amnesic shellfish poisoning mystery. Anal. Chem..

[115] Iverson F., Truelove J., Nera E., Tryphonas L., Campbell J., Lok E. (1989). Domoic acid poisoning and mussel-associated intoxication: Preliminary investigations into the response of mice and rats to toxic mussel extract. Food Chem. Toxicol..

[116] Trainer V.L., Bates S.S., Lundholm N., Thessen A.E., Cochlan W.P., Adams N.G., Trick C.G. (2012). *Pseudo-nitzschia* physiological ecology, phylogeny, toxicity, monitoring and impacts on ecosystem health. Harmful Algae.

[117] Takemoto T., Daigo K., Kondo Y., Kondo K. (1966). Studies on the constituents of *Chondria armata.* VIII. On the structure of domoic acid. Yakugaku Zasshi.

[118] Ohfune Y., Tomita M. (1982). Total synthesis of domic acid. A revision of original structure. J. Am. Chem. Soc..

[119] Bates S.S., Trainer V.L., Graneli E., Turner J.T. (2006). The ecology of harmful diatoms. Ecological Studies.

[120] Trainer V.L., Hickey B.M., Bates S.S., Walsh P.J., Smith S.L., Fleming L.E., Solo-Gabriele H., Gerwick W.H. (2008). Toxic diatoms. Ocean and Human Health: Risks and Remedies from the Sea.

[121] Lelong A., Hegaret H., Soudant P., Bates S.S. (2012). *Pseudo-nitzschia* (Bacillariophyceae) species, domoic acid and amnesic shellfish poisoning: Revisting previous paradigm. Phycologia.

[122] Kobayashi K., Takata Y., Kodama M. (2009). Direct contact between *Pseudo-nitzschia multiseries* and bacteria is necessary for the diatom to produce a high level of domoic acid. Fish. Sci..

[123] Pulido O.M. (2008). Domoic acid toxicologic pathology: A review. Mar. Drugs.

[124] Lefebvre K.A., Robertson A. (2010). Domoic acid and human exposure risks: A review. Toxicon.

[125] Hess P., Gallacher L.A., Bates S., Quilliam M.A. (2001). Determination and confirmation of amnesic shellfish poisoning toxin, domoic acid in shellfish from Scotland by liquid chromatography and mass spectrometry. J. AOAC Int..

[126] Kotaki Y., Koike K., Sato S., Ogata T., Fukuyo Y., Kodama M. (1999). Confirmation of domoic acid production of *Pseudo-nitzschia multiseries* isolated from Ofunato Bay, Japan. Toxicon.

[127] Amzil Z., Fresnel J., le G.D., Billard C. (2001). Domic acid accumulation in French shellfish in relation to toxic species of *Pseudo-nitzschia multiseries* and *P. pseudodelicatissima*. Toxicon.

[128] James K.J., Gillman M., Amandi M.F., Lopez-Rivera A., Puente P.F., Lehane M., Mitovic S., Furey A. (2005). Amnesic shellfish poisoning toxins in bivalve molluscs In Ireland. Toxicon.

[129] Scholin C.A., Gulland F., Doucette G.J., Benson S., Busman M., Chavez E.P., Cordaro J., DeLong R., de Vogelaere A., Harvey J. (2000). Mortality of sea lions along the central California coast linked to a toxic diatom bloom. Nature.

[130] Maeda M., Kodama T., Tanaka T., Yoshimuzu H., Takemoto T., Nomoto K., Fujita T. (1986). Structures of isodomoic acids A, B, and C, novel insecticidal amino acids from the red alga *Chondria armata*. Chem. Pharm. Bull..

[131] Wright J.L.C., Falk M., McInnes A.G., Walter J.A. (1990). Identification of isodomoic acid D and 2 new geometrical isomers of domoic acid in toxic mussels. Can. J. Chem..

[132] Clayden J., Read B., Hebdich K.R. (2005). Chemistry of domoic acid, isodomoic acids, and their analogues. Tetrahedron.

[133] Pearson M.L., Dortch Q., Turner R.E. (2002). Sedimental evidence of an increase in *Pseudo-nitzschia* (Bacillariophyceae) abundance in response to coastal etuterophication. Limnol. Oceanogr..

[134] Mos L. (2001). Domoic acid: A fascinating marine toxin. Environ. Toxicol. Pharmacol..

[135] Stonik I.V., Orlova T.Y., Lindholm N. (2011). Diversity of *Pseudo-nitzschia* from the western North Pacific. Diatom Res..

[136] Silver M.W., Bargu S., Coale S.L., Benitz-Nelson C.R., Garcia A.C., Roberts K.J., Sekula-Wood E., Bruland K.W., Coale K.H. (2010). Toxic diatoms and domoic acid in natural and iron-enriched waters of the oceanic Pacific. Proc. Natl. Acad. Sci. USA.

[137] Gillard J., Frenkel J., Devos V., Sabbe K., Paul C., Rempt M., Inze D., Pohnert G., Vuylsteke M., Vyverman W. (2013). Metabolomics enables the structure elucidation of a diatom sex pheromone. Angew. Chem. Int. Ed. Engl..

[138] Bucci M. (2013). Proline draws a diatom. Nat. Chem. Biol..

[139] Frenkel J., Wess C., Vyverman W., Pohnert G. (2014). Chiral separation of a diketopiperazine pheromone from marine diatoms using supercriticial fluid chromatography. J. Chromatogr. B Analyt. Technol. Biomed. Life Sci..

[140] Kroger N., Deitzmann R., Sumpler M. (1999). Polykationic peptides from diatom biosilica that direct silica nanosphere formation. Science.

[141] Sumper M. (2002). A phase separation model for the nanopatterning of diatom bioslica. Science.

[142] Poulsen N., Kroger N. (2004). Silica morphogenesis by alternative processing of silaffins in the diatom *Thalassiosira pseudonana*. J. Biol. Chem..

[143] Kroger N., Lorenz S., Brunner E., Sumper M. (2002). Self-assembly of highly phosporylated silaffins and their function in biosilica morphogenesis. Science.

[144] Poulsen N., Sumper M., Kroger N. (2003). Biosilica formation in diatoms. Characterization of native sillafin-2 and its role *in silica* morphogenesis. Proc. Natl. Acad. Sci. USA.

[145] Sumper M., Kroger N. (2004). Silica formation in diatoms: The function of long-chain polyamines and silaffines. J. Mater. Chem..

[146] Kroger N., Deutzmann R., Bergsdorf C., Sumper M. (2000). Species-specific polyamines from diatoms control silica morphology. Proc. Natl. Acad. Sci. USA.

[147] Vardi A., Thamatrakoln K., Bidle K.D., Falkowski P.G. (2008). Diatom genomes come of age. Genome Biol..

[148] Ravin N.V., Galachyantz Y.P., Mardanov A.V., Beketsky A.V., Petrova D.P., Sherbakova T.A., Zakharova Y.R., Likhoshway E.V., Skryabin K.G., Grachev M.A. (2010). Complete sequence of the mitochondrial genome of a diatom alga *Synedra acus* and comparative analysis of diatom mitochondrial genomes. Curr. Genet..

[149] Outdot-Le Secq M.P., Grimwood J., Shapiro H., Armburst E.V., Bowler C., Green B.R. (2007). Chloroplast genomes of the diatoms *Thalassiosira pseudonana* and *Phaeodactylum tricornutum*: Comparison with other plastid genomes of the red lineage. Mol. Genet. Genomics.

[150] Galachyants Y.P., Morozov A.A., Mardanov A.V., Beketsky A.V., Ravin N.V., Petrova D.P., Likhoshway E.V. (2012). Complete chloroplast genome sequence of freshwater araphid pennate diatom alga *Synedra acus* from Baikal Lake. Int. J. Biol..

[151] Khozin-Goldberg I., Cohen Z. (2011). Unraveling algal lipid metabolism: Recent advances in gene identification. Biochimie.

[152] Tonon T., Qing R.W., Harvey D., Li Y., Larson T.R., Graham I.A. (2005). Identification of a long-chain polyunsaturated fatty acid acyl-coenzyme A synthetase from the diatom *Thalassiosira pseudonana*. Plant Physiol..

[153] Li H.-Y., Lu Y., Zheng J.-W., Yang W.-D., Liu J.-S. (2014). Biochemical and genetic engineering of diatoms for polyunsaturated fatty acid biosynthesis. Mar. Drugs.

[154] Niu Y.F., Zhang M.H., Li D.W., Yang W.D., Liu J.S., Bai W.B., Li H.Y. (2013). Improvement of neutral lipid and polyunsaturated fatty acid biosynthesis by overexpressing a type 2 diacylglycerol acyltransferase in marine diatom *Phaeodactylum tricornutum*. Mar. Drugs.

[155] Bellou S., Baeshen M.N., Elazzazy A.M., Aggeli D., Sayegh F. (2014). Microalgal lipids biochemistry and biotechnological perspectives. Biotechnol. Adv..

[156] Zaslavskaya L.A., Lippmeier J.C., Shih C., Ehrhardt D., Grossman A.R., Apt K.E. (2001). Trophic conversion of an obligate photoautotrophic organism through metabolic engineering. Science.

[157] Michael A.J. (2011). Molecular machines encoded by bacterial-derived multi-domain gene fusions that potentially synthesize, *N*-methylate and transfer long chain polyamines in diatoms. FEBS Lett..

[158] Bertrand M. (2010). Carotenoid biosynthesis in diatoms. Photosynth. Res..

[159] Fabris M., Matthijs M., Carbonelle S., Moses T., Pollier J., Dasseville R., Baart G.J.E., Vyvermann W., Goossens A. (2014). Tracking the sterol biosynthesis pathway of the diatom. New Phytol..

[160] Boissonneault K.R., Henningsen B.M., Bates S.S., Robertson D.L., Milton S., Pelletier J., Hogan D.A., Housman D.E. (2013). Gene expression studies for the analysis of the marine diatom *Pseudo-nitzschia multiseries*. BMC Mol. Biol..

[161] Armbrust E.V., Berges J.A., Bowler C., Green B.R., Martinez D., Putnam N.H., Zhou S., Allen A.E., Apt K.E., Bechner M. (2004). The genome of the diatom Thalassiosira pseudonana: Ecology, evolution, and metabolism. Science.

[162] Smith S.R., Abbriano R.M., Hildebrand M. (2012). Comparative analysis of diatom genomes reveals substantial differences in the organization of carbon partitioning pathways. Algal Res..

